# Analysis of the plasmid-based ts-mutant *Δpa0947* (pTS-*pa0947*) reveals *pa0947* essentiality and *dnaA*-centered suppressor bypass in *Pseudomonas aeruginosa*

**DOI:** 10.1128/spectrum.00619-26

**Published:** 2026-09-22

**Authors:** Qinghai Tang, Huiting Zhu, Zhili Yang

**Affiliations:** 1 Systems Biology Laboratory, School of Marine Science and Technology, Zhejiang Ocean University 71233, Zhoushan, Zhejiang, China; University at Albany, Albany, New York, USA

**Keywords:** plasmid-based ts-allele, essential gene, suppressor mutation, *Pseudomonas aeruginosa*

## Abstract

PA0947 is annotated as an essential hypothetical protein in *Pseudomonas aeruginosa* and shares substantial sequence identity with the *Escherichia coli* replication regulator Hda, yet its cellular function and genetic bypass logic in *P. aeruginosa* have not been experimentally characterized. Here, we constructed a plasmid-based temperature-sensitive complementation strain, *Δpa0947*(pTS-*pa0947*), to enable conditional depletion of *pa0947*. The mutant grew like wild type at 30°C but failed to proliferate at 42°C, demonstrating *pa0947* as essential for growth. *pa0947* depletion triggered rapid filamentation, increased PicoGreen-associated fluorescence, and an elevated oriC/ter ratio, consistent with dysregulated replication initiation. In addition, *E. coli hda* did not complement *pa0947* depletion, and overexpression of either *pa0947* or *E. coli hda* impaired growth, indicating species-dependent incompatibility and dosage sensitivity. To identify suppressors capable of bypassing *pa0947* loss, we performed a spontaneous suppressor screen and isolated four independent suppressor mutants that grew without *pa0947*. Genome resequencing of these suppressors revealed convergent mutations in the *dnaA* locus, including upstream regulatory indels and C-terminal coding changes. Upstream mutations reduced *dnaA* transcript levels by qRT-PCR, whereas coding changes compromised DnaA–oriC binding in EMSA. Together, these results identify *dnaA* as the convergent bypass locus for *pa0947* essentiality and link *pa0947* function to tight control of DnaA-driven initiation in *P. aeruginosa*.

**IMPORTANCE:**

Many bacterial essential genes remain poorly understood, limiting efforts to exploit them as antibiotic targets. PA0947 is predicted to be essential in the opportunistic pathogen *P. aeruginosa*, yet its essentiality and physiological role have not been experimentally defined. Using a conditional depletion strategy based on plasmid-borne, temperature-sensitive complementation, we show that PA0947 loss causes severe growth failure, filamentation, and abnormal DNA accumulation, consistent with disrupted control of chromosome duplication. Suppressor analysis identified four independent suppressors, including promoter lesions that reduced dnaA expression and coding changes that compromised DnaA–oriC binding. Together, our results establish in *P. aeruginosa* that PA0947 is required to keep replication initiation within a narrow activity window compatible with robust growth, and they highlight conditional depletion plus suppressor mapping as a broadly useful strategy to uncover the function and bypass logic of essential bacterial genes.

## INTRODUCTION

*Pseudomonas aeruginosa* is a Gram-negative opportunistic pathogen that thrives in diverse environments and causes difficult-to-treat infections in immunocompromised patients (1). Its broad intrinsic and acquired resistance underscores the need to identify new antibacterial targets beyond classical pathways (2, 3).

A productive route is to focus on essential genes whose inactivation is incompatible with growth since their products anchor core cellular processes and often reveal druggable vulnerabilities (4–6). Transposon-based surveys in *P. aeruginosa* have defined a few hundred essentials, and a notable subset encode hypothetical or poorly characterized proteins (7). These “unknown essentials” are attractive: they are required for viability and, when functionally resolved, can uncover fundamental biology while expanding the antibacterial target space (5, 8, 9).

PA0947, previously annotated as an essential hypothetical protein (7), was shown in this study to share approximately 46.8% amino acid identity with the Hda protein from *E. coli*, supporting its designation as an Hda homolog in *P. aeruginosa* (10). Hda is an ATPase associated with diverse cellular activities (AAA^+^) factor that couples to the DNA polymerase sliding clamp to accelerate ATP hydrolysis by DnaA, the initiator of chromosome replication (11–14). This regulatory inactivation of DnaA (RIDA) converts active DnaA–ATP to inactive DnaA–ADP and enforces the “once-and-only-once” rule for replication initiation (15, 16).

Many essential genes are suppressible, with viability restored by second-site mutations that rewire the affected pathway (17, 18). In *E. coli*, loss of *hda* disrupts RIDA and drives replication overinitiation accompanied by severe growth impairment, but growth can be alleviated by suppressor mutations that attenuate DnaA activity (19). This finding underscores a tightly regulated interplay between Hda and DnaA that ensures replication initiates only once per cell cycle. However, whether *pa0947* can be bypassed by suppressor mutations in *P. aeruginosa*—and, if so, whether suppression converges on tuning DnaA activity as in *E. coli*—remains unknown because PA0947’s cellular function has not been experimentally characterized in this organism.

To address this gap, we constructed a conditional mutant strain *Δpa0947*(pTS-*pa0947*) (hereafter, PA0947-ts) by deleting the chromosomal *pa0947* and complementing it with a temperature-sensitive (ts) plasmid expressing *pa0947* under its native promoter, following our previously established approach (20–23). This system enabled controlled *pa0947* depletion and phenotypic analysis at the restrictive temperature of 42°C. Using suppressor screening (20, 21, 23) and genome resequencing, we found that bypass mutations converged on the *dnaA* locus, affecting either *dnaA* expression or DnaA–oriC binding. Together, our findings demonstrate PA0947 as an essential Hda homolog in *P. aeruginosa* and link its essentiality to tight control of DnaA-dependent initiation.

## RESULTS

### Construction of PA0947-ts and validation of its growth defect in *P. aeruginosa*

**Fig 1:**
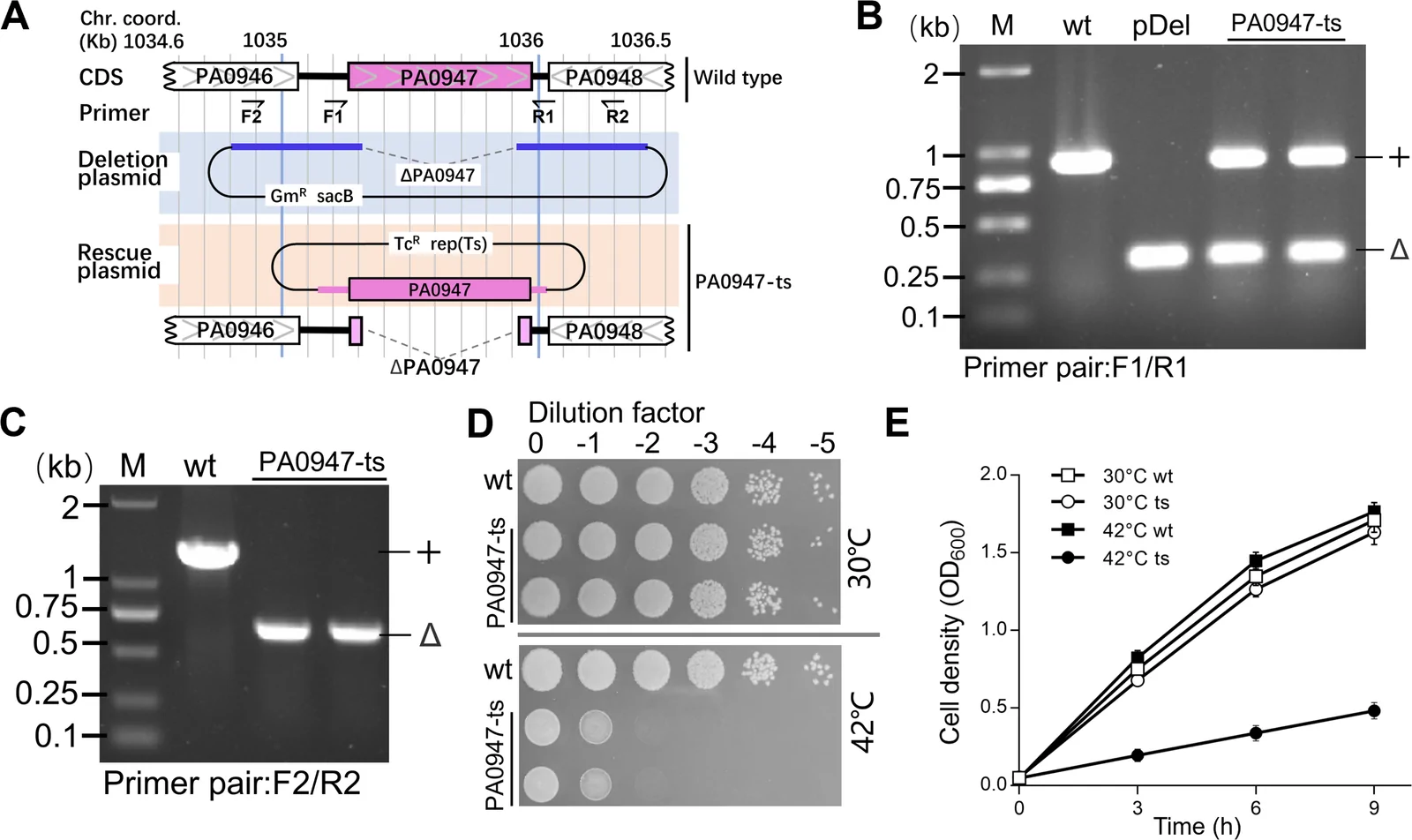
Construction and characterization of the *pa0947* ts mutant PA0947-ts. (**A**) Schematic of the locus and plasmid strategy. The chromosomal region flanking *pa0947* is shown with primer sites F1/R1 and F2/R2. The deletion plasmid carries the *Δpa0947* allele (Gm^R^, *sacB*), and the ts rescue plasmid (pTS-*pa0947*) carries a native-promoter *pa0947* allele (Tc^R^, *rep*^ts^). The resulting strain harbors a chromosomal *Δpa0947* together with a complementing *pa0947* on the ts plasmid. (**B and C**) PCR analysis of *pa0947* alleles using primer pairs F1/R1 and F2/R2 to distinguish the *pa0947^+^* and chromosomal *Δpa0947* alleles in PA0947-ts (ts) strains. (**D**) Spot-plating assay comparing wt and ts at 30°C and 42°C. (**E**) Growth curves of wt and ts at 30°C and 42°C. Cell density (OD_600_) was recorded over 9 h; data are mean ± SD (*n* = 3).

To test whether *pa0947* is essential for growth in *P. aeruginosa*, we generated a plasmid-based temperature-sensitive mutant, PA0947-ts (ts), following our previously established three-step workflow (20–23). The design places a chromosomal *Δpa0947* deletion together with a rescue copy of *pa0947* carried on a temperature-sensitive plasmid under the native promoter (Fig. 1A). PCR assays using primer pair F1/R1 detected the expected products: the deletion plasmid control (pDel) lacked the wild-type (wt) band, while the PA0947-ts strain yielded both wt-sized and deletion-sized fragments, consistent with coexistence of the plasmid-borne *pa0947^+^* and the chromosomal *Δpa0947* allele (Fig. 1B). A second PCR with F2/R2, positioned outside the complementing region, verified that the chromosome of the PA0947-ts strain carries the *Δpa0947* allele (Δ band), whereas the wt control shows only the *pa0947^+^* band (Fig. 1C). Phenotypically, spot-plating revealed that PA0947-ts grows comparably to wt at 30°C but is strongly impaired at 42°C, indicating a restrictive temperature-dependent defect (Fig. 1D). Growth-curve measurements corroborated the plating results: the PA0947-ts strain exhibited near-wt growth at 30°C yet failed to proliferate at 42°C (Fig. 1E), further supporting that *pa0947* is essential for *P. aeruginosa* growth.

### *E. coli* Hda does not complement PA0947 deficiency and is detrimental when overexpressed

**Fig 2:**
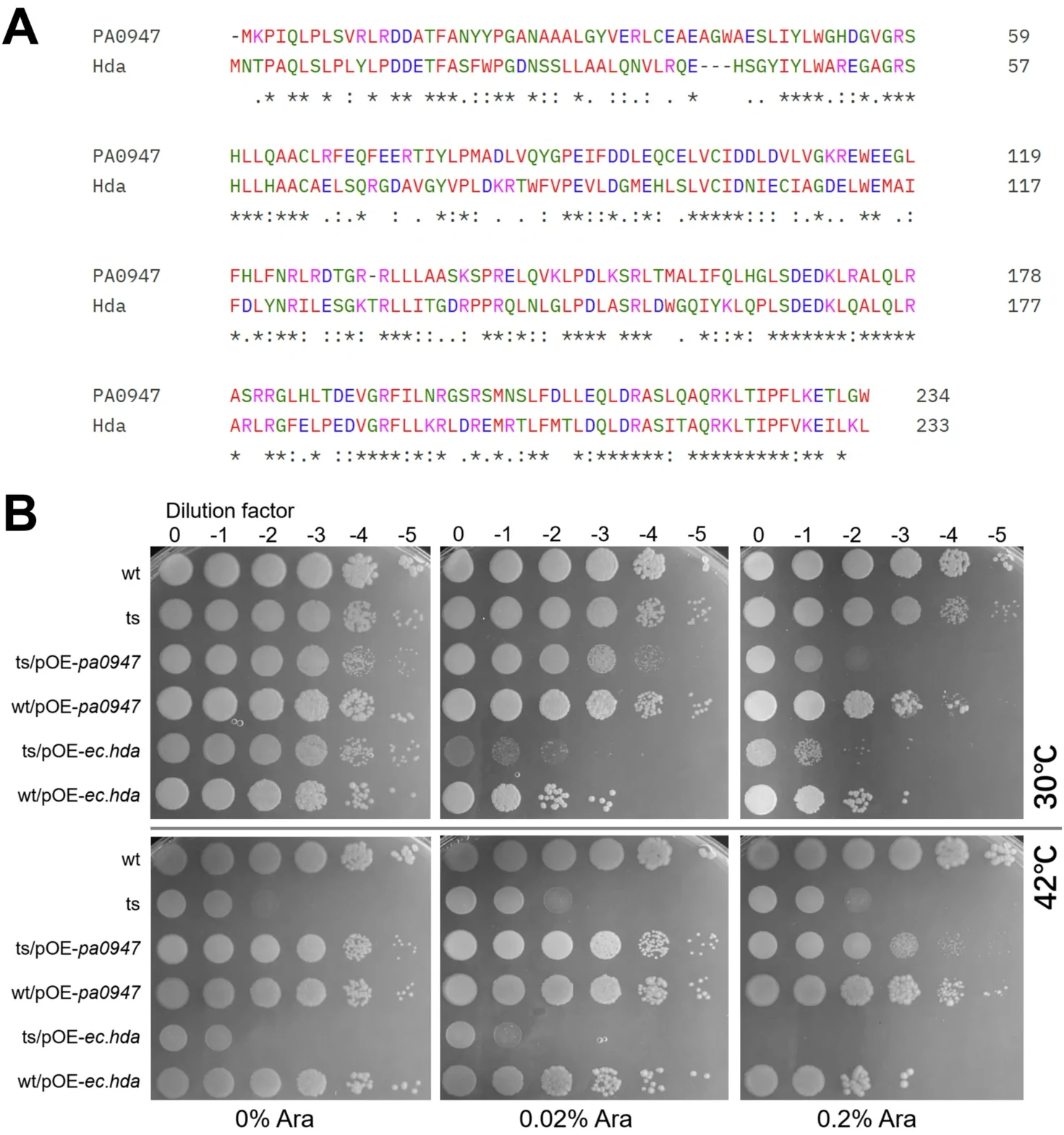
*E. coli* Hda does not complement PA0947, and overexpression is detrimental. (**A**) Protein sequence alignment between *P. aeruginosa* PA0947 and *E. coli* Hda. Symbols: “*” identical; “:” strongly similar; “.” weakly similar. (**B**) Spot-plating assays of wt*,* PA0947-ts (ts), ts/pOE-*pa0947*, wt/pOE-*pa0947*, ts/pOE-*ec.hda*, and wt/pOE-*ec.hda* at 30°C (top panel) and 42°C (bottom panel) under 0%, 0.02%, or 0.2% arabinose.

Using pairwise protein sequence alignment, we found that PA0947 from *P. aeruginosa* and Hda from *E. coli* exhibit 461% sequence identity across 233 aligned amino acid residues (Fig. 2A). This degree of conservation, which exceeds the commonly applied 40% threshold, supports the notion that the two proteins are likely to share a similar structural and potentially related biochemical functions (10).

To test whether the *E. coli* ortholog Hda functionally complements PA0947 in *P. aeruginosa*, we constructed arabinose-inducible overexpression plasmids pOE-*pa0947* and pOE-*ec.hda* by cloning each gene under the control of the P_BAD_ promoter (24) in the broad-host-range pBBR1MCS-5 plasmid (25) and compared growth by spot-plating at 30°C (permissive) and 42°C (restrictive) under 0%, 0.02%, and 0.2% arabinose. At 30°C, the PA0947-ts strain behaved like the wild type (wt), and introducing pOE-*pa0947* or pOE-*ec.hda* had little effect without inducer (Fig. 2B); strong induction (0.2% arabinose) reduced colony formation to varying degrees in both wt and ts backgrounds (Fig. 2B). At 42°C, the ts strain showed a severe growth defect, whereas introduction of pOE-*pa0947* restored growth at 0% and 0.02% arabinose (leaky/mild expression), but high-level expression (0.2% arabinose) impaired growth in both ts/pOE-*pa0947* and wt/pOE-*pa0947* (Fig. 2B). In contrast, pOE-*ec.hda* failed to rescue the *pa0947* ts strain at 42°C at any inducer level, and its overexpression (0.2% arabinose) markedly inhibited growth in both ts and wt backgrounds (Fig. 2B). These data indicate that *E. coli* Hda cannot functionally substitute for PA0947 in *P. aeruginosa*, implying species-specific incompatibility of Hda-dependent interactions. In addition, overexpression of either PA0947 or *E. coli* Hda reduced colony formation, suggesting dosage-dependent toxicity in *P. aeruginosa*.

### *pa0947* is required for normal cell morphology and replication-associated DNA profiles in *P. aeruginosa*

**Fig 3:**
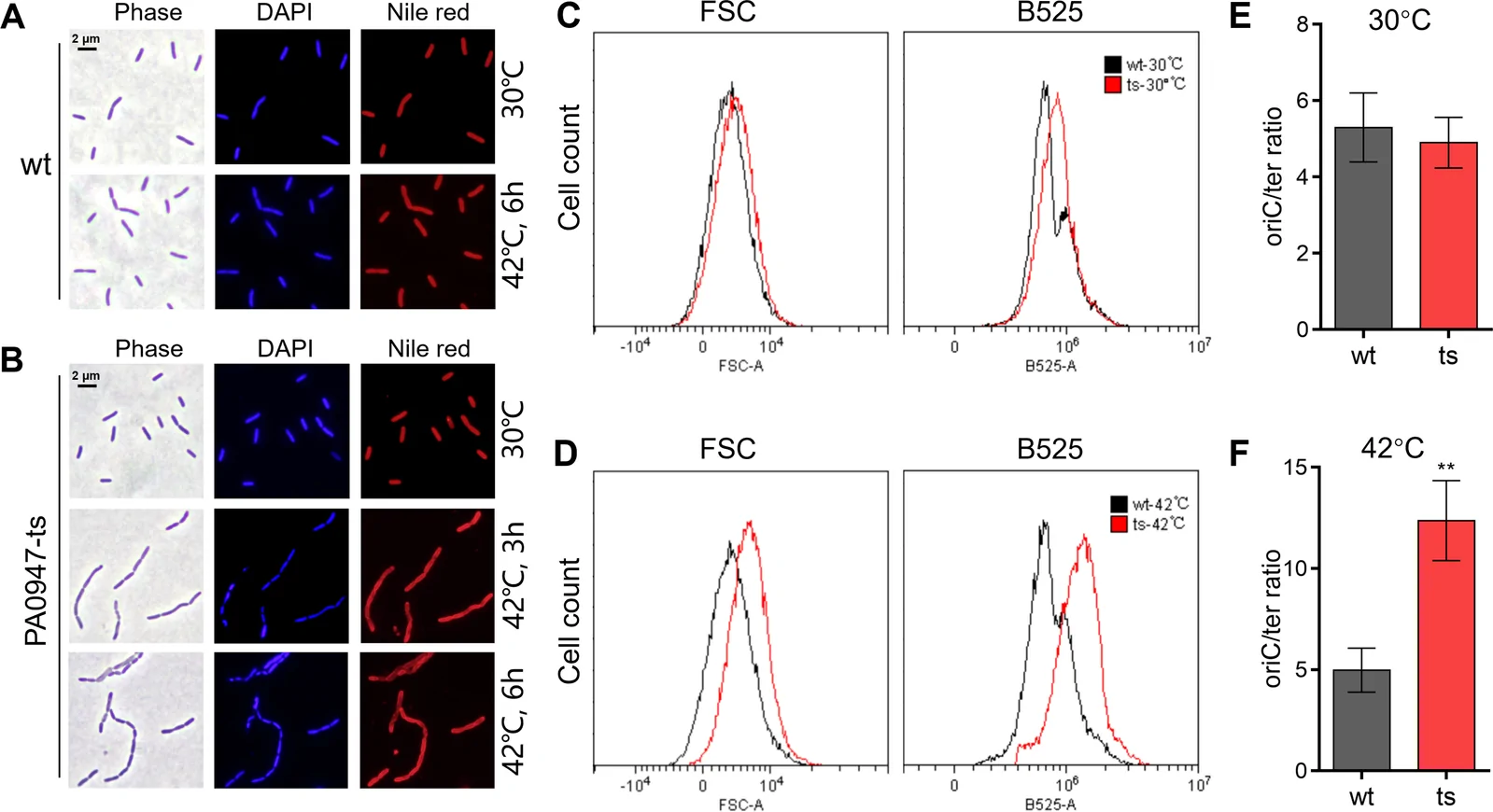
*pa0947* depletion causes filamentation and is associated with increased PicoGreen fluorescence and an elevated oriC/ter ratio at 42°C. (**A**) Wild-type morphology at 30°C and after 6 h at 42°C. Scale bar, 2 μm. (**B**) Morphology of PA0947-ts (ts) at 30°C (top), after 3 h at 42°C (middle), and 6 h at 42°C (bottom). Phase-contrast images are shown alongside fluorescence images stained with DAPI (DNA, blue) and Nile red (membrane, red). (**C and D**) Flow-cytometric overlays of FSC-A and PicoGreen fluorescence (B525-A) for wt and ts at 30°C (**C**) or after incubation at 42°C for 3 h (**D**). (**E and F**) qPCR-based oriC/ter analysis at 30°C (**E**) and 42°C (**F**). Data are mean ± SD (*n* = 3). **, *P* < 0.01.

To determine the terminal phenotype of PA0947-ts under *pa0947*-depletion conditions, we examined cell morphology and DNA-associated fluorescence under permissive (30°C) and restrictive conditions (42°C, where *pa0947* function is progressively lost in the ts strain). Wild-type cells remained short rods at 30°C and showed no obvious changes after incubation at 42°C (Fig. 3A). The ts strain was indistinguishable from wild type at 30°C (Fig. 3B, top row). However, upon incubation at 42°C, ts cells became progressively elongated within 3 h and frequently formed filaments/linear chains with incomplete constrictions (Fig. 3B, middle row). By 6 h, phase-pale “ghost” cells and lysed cells were also observed, indicative of cell death (Fig. 3B, bottom row). We next examined cellular DNA-associated fluorescence by flow cytometry after PicoGreen staining (26, 27). At 30°C, wild-type and ts populations showed largely overlapping FSC profiles and B525 fluorescence distributions (Fig. 3C). In contrast, after incubation at 42°C for 3 h, the ts population exhibited a right shift in FSC, consistent with increased cell size/elongation, and a clear right shift in PicoGreen fluorescence (B525) relative to wild type (Fig. 3D); median B525-A values from replicate FACS analyses are summarized in Fig. S1.

To further assess whether PA0947 depletion affected replication status, we measured the oriC/ter ratio by qPCR. At 30°C, the oriC/ter ratio of PA0947-ts was not increased compared with wt cells (Fig. 3E). In contrast, after incubation at 42°C, PA0947-ts showed a markedly increased oriC/ter ratio compared with wt cells grown under the same condition (Fig. 3F). These results indicate increased origin-proximal DNA abundance following PA0947 depletion.

### Isolation and characterization of four suppressors that bypass *pa0947* depletion

**Fig 4:**
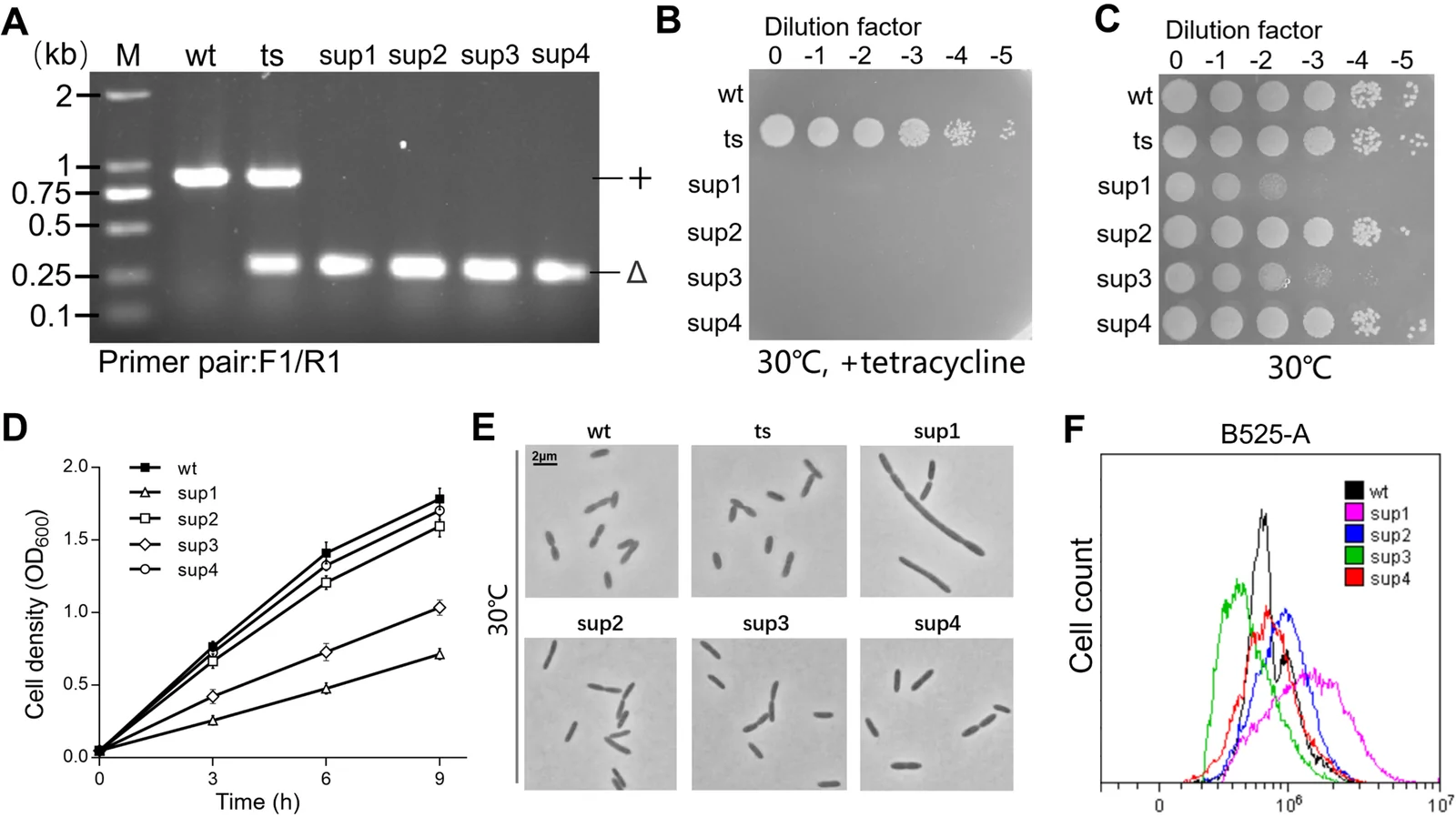
Isolation and phenotypic analysis of suppressors derived from the PA0947-ts strain. (**A**) PCR verification using primer pair F1/R1. wt shows only the *pa0947^+^* band; PA0947-ts (ts) shows both + and Δ bands; sup1–sup4 show only the Δ band, confirming loss of the chromosomal wt allele. (**B**) Tetracycline validation at 30°C. On LB–Tet plates, ts (Tet^R^ plasmid present) grows, whereas sup1–sup4 are Tet-sensitive, indicating loss of the ts rescue plasmid. (**C**) Spot-plating on LB at 30°C of wt and suppressor strains (sup1–sup4). (**D**) Growth curves at 30°C of wt and suppressor strains. (**E**) Phase-contrast microscopy of wt and suppressor strains grown at 30°C. Scale bar, 2 µm. (**F**) Flow-cytometric histograms of PicoGreen fluorescence for wt and suppressor strains grown in LB medium at 30°C to logarithmic phase.

To uncover second-site mutations that alleviate the growth defect caused by *pa0947* deletion, we performed a spontaneous suppressor screen from the PA0947-ts background, as previously described (20, 21, 23), and isolated four independent suppressor clones (designated sup1–sup4). PCR with primer pair F1/R1 (Fig. 1A) showed that all four suppressors carried only the chromosomal *Δpa0947* allele and lacked the wild-type band, whereas the PA0947-ts displayed both the wt-sized and Δ-sized amplicons as expected (Fig. 4A). Consistently, on LB–tetracycline plates at 30 °C, the PA0947-ts grew because it retained the Tet^R^ pTS-*pa0947* rescue plasmid, whereas sup1–sup4 failed to form colonies, confirming that the suppressor isolates had lost the ts rescue plasmid and could grow without the plasmid-borne *pa0947* copy (Fig. 4B).

Phenotypically, spot-plating at 30°C revealed that sup2 and sup4 recovered near-wt growth, whereas sup1 and sup3 exhibited growth-attenuated (Fig. 4C). Growth-curve measurements corroborated these trends (Fig. 4D). Microscopy at 30°C further showed that sup1 cells frequently appeared as filaments/linear chains with incomplete constrictions, while sup2, sup3, and sup4 largely returned to short rod-shaped morphology comparable to wild type (Fig. 4E). Consistent with these microscopic observations, FSC-A analysis showed a clear rightward shift for sup1, whereas sup2, sup3, and sup4 displayed FSC-A profiles broadly similar to wt, with only modest shifts (Fig. S2A). These data indicate that sup1 retained a pronounced cell-size/morphology defect despite bypassing *pa0947* growth defect.

We next examined PicoGreen-associated B525-A fluorescence. In representative histograms, sup4 closely overlapped with wt, sup2 displayed a modest rightward shift, sup3 shifted leftward, and sup1 showed a broadened, overall right-shifted profile (Fig. 4F); median B525-A values from replicate FACS analyses are summarized in Fig. S2B. Because PicoGreen-associated fluorescence can be influenced by cell size and event structure, we further measured oriC/ter ratios by qPCR. sup3 showed the lowest oriC/ter ratio, while sup2 and sup4 showed numerically reduced ratios, and sup1 remained close to wt (Fig. S2C). These data suggest that the suppressors tune DnaA-dependent initiation to different extents, with sup3 showing the strongest reduction in origin-proximal marker frequency. The major genetic changes and associated phenotypic features of each suppressor are summarized in Table S1.

Together, these results show that distinct suppressor classes can bypass *pa0947* depletion, with variable restoration of growth, morphology, DNA-associated fluorescence, and replication-associated profiles toward wild-type levels.

### Mutation of *dnaA* identified as a suppressor of *Δpa0947* growth defect

**Fig 5:**
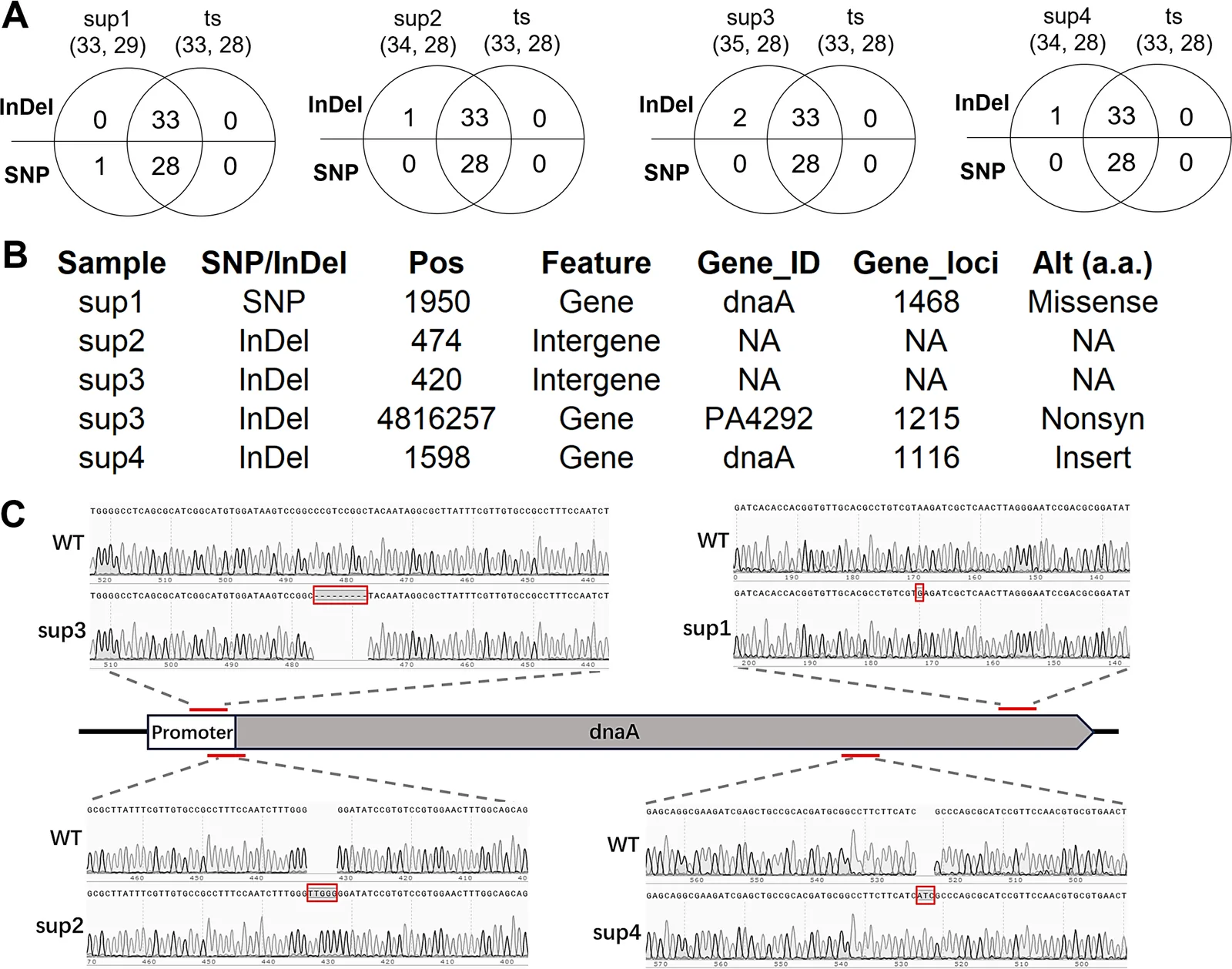
Genome resequencing identifies *dnaA* mutations in PA0947-ts derived suppressors, with Sanger confirmation. (**A**) Summary of SNPs and indels in sup1–sup4 relative to the parental PA0947-ts (ts) strain. Venn diagrams show variants shared with, or unique to, each suppressor. (**B**) Suppressor-specific variants identified by genome resequencing. (**C**) Sanger sequencing confirmation of the *dnaA*-locus variants detected by genome resequencing.

To define the mutations responsible for suppression, we performed genome resequencing of sup1–sup4 and the parental PA0947-ts strain (see Materials and Methods). Across all four isolates, the vast majority of SNPs/indels were shared with the parental background, whereas each suppressor carried a small set of unique variants (Fig. 5A). Among these, all four suppressors harbored mutations in the *dnaA* locus (Fig. 5B and C and Table S2). Specifically, sup1 and sup4 carried coding-sequence changes in the C-terminal region of DnaA (sup1, c.1468A > G, p.Lys490Glu; sup4, an in-frame ATC insertion adding one Ile residue), whereas sup2 and sup3 harbored indels in the *dnaA* upstream regulatory region (sup2, −9_−8insTTGGG; sup3, −62_−54delCCGTCCGGC; positions relative to the GTG start codon) (Fig. 5C). In addition, sup3 also carried an additional mutation in *pa4292*, a nonessential gene annotated as a probable phosphate transporter (Fig. 5B and Table S2) and reported as the *P. aeruginosa* PitA homolog (28). Because this variant was not shared by the other suppressors, we focused on *dnaA* as the common suppressor locus. Sanger sequencing confirmed the upstream regulatory region indels in sup2/sup3-*dnaA* and the C-terminal coding changes in sup1/sup4-*dnaA* (Fig. 5C). These suppressor-specific genetic changes, together with the corresponding growth, morphology, PicoGreen fluorescence, qRT-PCR, and electrophoretic mobility shift assay (EMSA) phenotypes, are summarized in Table S1. Together, these data implicate *dnaA* as the common suppressor locus, and the locations of the mutations suggest that they may alter DnaA dosage or function.

### Upstream regulatory and coding suppressor mutations tune *dnaA* expression and DnaA engagement with oriC

**Fig 6:**
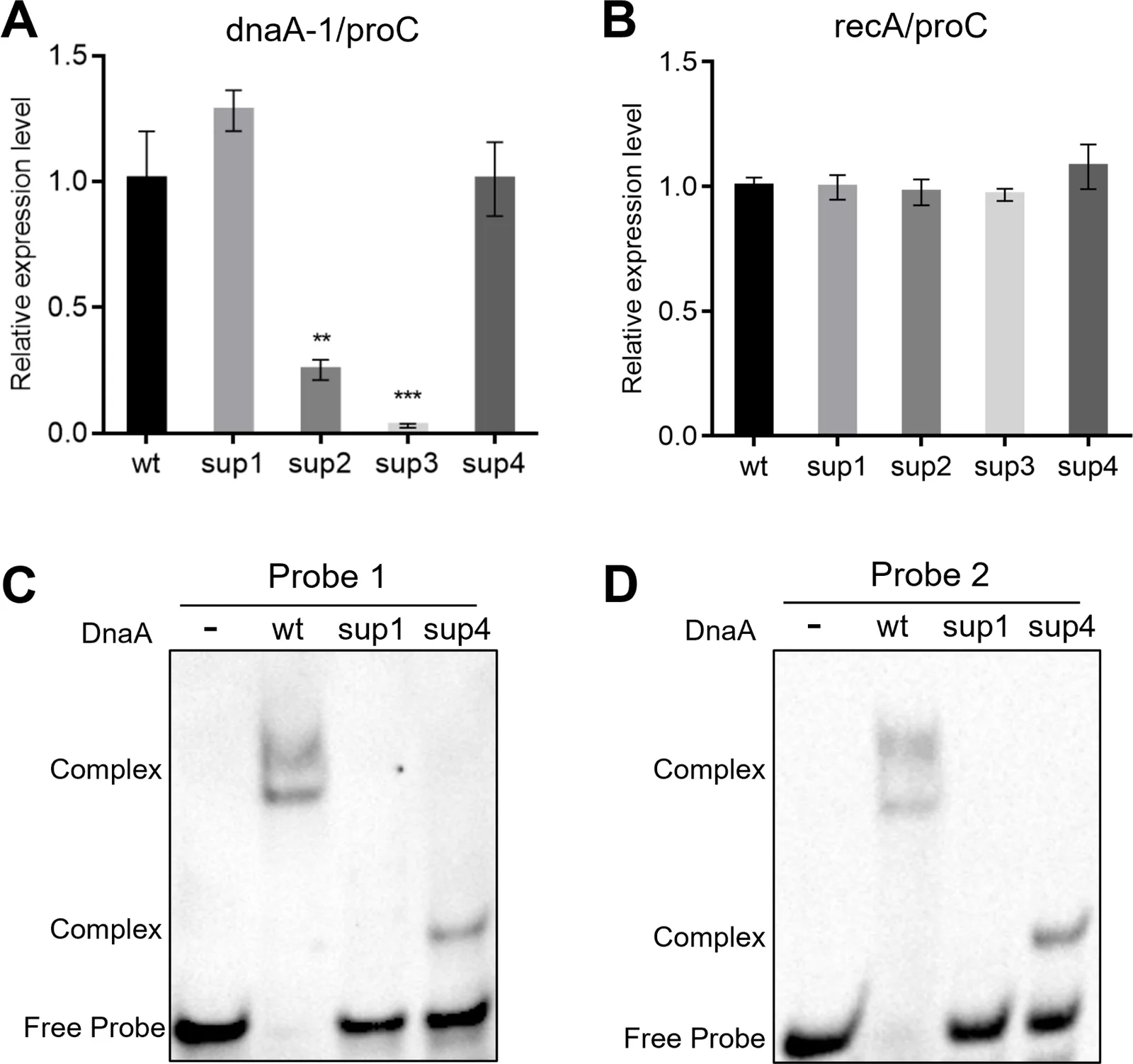
Suppressor mutations in *dnaA* reduce *dnaA* expression or compromise DnaA–oriC binding. (**A**) *dnaA* transcript levels in wt and sup1–sup4 measured by qRT-PCR with the dnaA-1 primer set and normalized to *proC*. (**B**) *recA* transcript levels in wt and sup1–sup4 normalized to *proC*. Data are mean ± SD (*n* = 3). **, *P* < 0.01; ***, *P* < 0.001 (vs wt). (**C and D**) EMSA of purified His-tagged DnaA variants (wt, sup1, sup4) with two oriC-derived duplex probes each containing a single high-affinity DnaA box (Table 1). Positions of protein–DNA complexes and free probe are indicated.

As identified by genome resequencing (Fig. 5), sup2/sup3 carry upstream regulatory indels, whereas sup1/sup4 harbor *dnaA* coding changes. To quantify the impact of the upstream regulatory indels, we measured *dnaA* transcript levels by qRT-PCR using two independent primer sets (*dnaA*-1 and *dnaA*-2; amplicons positioned away from the mutation sites) (Table 1) and normalized to the housekeeping gene *proC* (29). Using the dnaA-1 primer set, *dnaA* transcript levels in sup2 were reduced to ~25% of the wt level, whereas those in sup3 were reduced to <5% of wt (Fig. 6A). In contrast, *dnaA* transcript levels in sup1 and sup4 remained near wt (Fig. 6A). A similar trend was obtained with the second primer set, dnaA-2, and is shown in Fig. S3. In addition, *recA* (30) expression normalized to *proC* remained largely unchanged among wt and the suppressor strains, serving as a control gene whose expression was not substantially affected (Fig. 6B).

**TABLE 1:** Oligonucleotides, plasmids, and strains used in this study

Name	Sequence (5′−3′) or relevant genotype	Usage or source/reference
Oligonucleotides		
F1	TGCTTGCCTAGTTGCTCATTGTG	Assay pa0947 alleles in chr. and ts-plasmid
R1	ACGGATAGAAGGCGGTCACTTC	Assay pa0947 alleles in chr. and ts-plasmid
F2	GTTGCAACGCCTGCTCGATC	Assay pa0947 alleles in chr. but not ts-plasmid
R2	CTGGGCCTGCTACGTGGTCA	Assay pa0947 alleles in chr. but not ts-plasmid
dnaA-1-qPCR-F	CAACCTCAAGCACGGCTACA	Quantify dnaA mRNA (qRT-PCR)
dnaA-1-qPCR-R	GAAACGTTCCGAATGCAGGT	Quantify dnaA mRNA (qRT-PCR)
dnaA-2-qPCR-F	TTGCAGCTCAACGCCATCAA	Quantify dnaA mRNA (qRT-PCR)
dnaA-2-qPCR-R	GCTGGTGAGGATCACCTGCT	Quantify dnaA mRNA (qRT-PCR)
recA-qPCR-F	GCAAGATCACCGGCAATATC	qRT-PCR housekeeping control (recA)
recA-qPCR-R	TTACCGCCGGTGGTGGTTTC	qRT-PCR housekeeping control (recA)
proC-qPCR-F	CAGGCCGGGCAGTTGCTGTC	qRT-PCR housekeeping control (proC)
proC-qPCR-R	GGTCAGGCGCGAGGCTGTCT	qRT-PCR housekeeping control (proC)
oriC-qPCR-F	GAAGCCTGGGGATGAACGAG	qPCR-based oriC/ter analysis
oriC-qPCR-R	GGTCGTCCTGGACTTTGTGG	qPCR-based oriC/ter analysis
ter-qPCR-F	TGCGCATCTGTTCTTCAGAGT	qPCR-based oriC/ter analysis
ter-qPCR-R	CTATCCTGGCAAGTGGGGTC	qPCR-based oriC/ter analysis
dnaA-pet28a-F	cagcaaatgggtcgcggatccTCCGTGGAACTTTGGCAGCA	Clone dnaA for His-tagged expression
dnaA-pet28a-R	tgcggccgcaagcttgtcgacGGTTGTCAGGGTACGCAGCA	Clone dnaA for His-tagged expression
Probe-1-F	tgcgcagggatgtacccccttcaaagcaaggg**ttatccaca**aagtccaggacgaccgtccgtcggcctgc	EMSA probe oligos^*a*^
Probe-1-R	gcaggccgacggacggtcgtcctggactt**tgtggataa**cccttgctttgaagggggtacatccctgcgca	EMSA probe oligos^*a*^
Probe-2-F	aagcgcctattgtagccggacgggccggac**ttatccaca**tgccgatgcgctgaggccccaagcaaatcaagaccttaata	EMSA probe oligos^*a*^
Probe-2-R	tattaaggtcttgatttgcttggggcctcagcgcatcggca**tgtggataa**gtccggcccgtccggctacaataggcgctt	EMSA probe oligos^*a*^
mut-Probe-1-F	tgcgcagggatgtacccccttcaaagcaaggg**gcgaggtgc**aagtccaggacgaccgtccgtcggcctgc	EMSA specificity control probe oligos^*b*^
mut-Probe-1-R	gcaggccgacggacggtcgtcctggactt**gcacctcgc**cccttgctttgaagggggtacatccctgcgca	EMSA specificity control probe oligos^*b*^
Plasmids		
pDel	pUC-Gm^R^-sacB	(20–23)
pTS	pUC-Tc^R^-ori^ts^	(20–23)
pOE	pBBRMCS-5-araC-P_BAD_-Gm^R^	(20–23)
pDel-pa0947	pa0947 deletion cassette in pDel	This study
pTS-pa0947	pa0947 rescue cassette in pTS	This study
pOE-pa0947	araC-P_BAD_-pa0947 in pOE	This study
pOE-ec.hda	araC-P_BAD_-ec.hda in pOE	This study
pET28a-dnaA	T7-dnaA in pET28a	This study
Strains		
PAO1	Wild type	(20–23)
Δpa0947(pTS-pa0947)	pTS-pa0947 in Δpa0947	This study
sup1	Suppressor of Δpa0947	This study
sup2	Suppressor of Δpa0947	This study
sup3	Suppressor of Δpa0947	This study
sup4	Suppressor of Δpa0947	This study

^*a*^ DnaA box bold/underlined.

^*b*^ Mutated DnaA box, bold/underlined.

We next tested whether the coding changes in *dnaA* alter origin recognition. EMSA was performed with purified His-tagged DnaA variants (wt-DnaA, sup1-DnaA, and sup4-DnaA), whose expression and Ni-affinity purification were examined by Coomassie-stained SDS–PAGE and anti-His western blotting (Fig. S4), using two oriC-derived duplex probes (probe 1 and probe 2), each containing a single high-affinity DnaA box (shown in bold and underlined in Table 1) (31). With either probe, wt-DnaA produced robust mobility shifts with near-complete depletion of free probe and yielded two discrete shifted species (Fig. 6C and D). In contrast, sup1-DnaA showed no readily detectable shifted species under these conditions, and the free-probe band remained predominant (Fig. 6C and D). sup4-DnaA generated a single shifted band that migrated faster than the wt shifted species and left a substantial fraction of free probe unbound (Fig. 6C and D). The concordant patterns observed with probe 1 and probe 2 indicate that these defects reflect intrinsic properties of the DnaA variants rather than probe-specific effects (Fig. 6C and D). As a specificity control, wt DnaA shifted wild-type probe 1 but not mut-probe 1, in which the DnaA-box sequence was mutated, confirming specific DnaA-box recognition (Fig. S5). Together, these data suggest that suppression converges on attenuating DnaA function, either by reducing *dnaA* expression (sup2/sup3) or by compromising oriC binding (sup1/sup4).

## DISCUSSION

*pa0947* is an essential gene in *P. aeruginosa*, encoding the homolog of the replication regulatory factor Hda identified in *E. coli* (Fig. 2A) (7, 11, 32). Using a conditional temperature-sensitive allele, we confirmed that *P. aeruginosa* cannot survive without *pa0947* (Fig. 1), consistent with transposon-based essentiality data (7). Cells depleted of *pa0947* became highly filamentous and displayed increased PicoGreen-associated DNA fluorescence (Fig. 3) although the FACS profiles were interpreted cautiously because elongation and chain formation can affect fluorescence per flow-cytometry event. Importantly, qPCR-based oriC/ter analysis further showed an elevated origin-proximal marker frequency after *pa0947* depletion at 42°C (Fig. 3E and F), providing independent support for dysregulated replication initiation (11, 15). This phenotype parallels the over-initiation and filamentation reported for *E. coli Δhda* backgrounds, which are typically viable but prone to deregulated initiation and genomic instability (33, 34). Together, these observations support a model in which PA0947, like Hda, helps restrain the replication initiator DnaA to maintain proper once-per-cell-cycle control of replication initiation.

In *E. coli*, Hda-mediated RIDA promotes hydrolysis of ATP bound to DnaA immediately after replication initiation, thereby converting DnaA into an inactive ADP-bound form (35, 36). This interaction, which requires the Hda protein complexed with the DNA-loaded β sliding clamp, is essential for preventing extra initiations within a single cell cycle (11, 35). The pronounced filamentation, increased DNA-associated fluorescence, and elevated oriC/ter ratio upon *pa0947* depletion (Fig. 3) suggest that *P. aeruginosa* employs an Hda-dependent mechanism to limit DnaA activity after initiation. RIDA-like control appears broadly conserved among bacteria encoding Hda homologs (36, 37). Notably, whereas *E. coli Δhda* mutants are generally recoverable, they frequently exhibit abnormal initiation control and genetic instability (38). In contrast, consistent with prior transposon-insertion essentiality calls for *pa0947* in *P. aeruginosa* (7), under the experimental conditions used in this study, viable *pa0947*-null mutants were recovered only when suppressor mutations arose, indicating a stricter dependence on this function for robust growth in *P. aeruginosa*. This stricter dependence could arise from fewer redundant layers that constrain DnaA activity and heightened vulnerability to replication stress in *P. aeruginosa*. For example, SeqA-mediated origin sequestration provides an additional brake against premature re-initiation in *E. coli* (39), whereas SeqA orthologs do not appear to be present in Pseudomonads, including *P. aeruginosa* (40), potentially placing greater reliance on Hda-mediated RIDA to prevent over-initiation.

We also found that *hda* is not readily interchangeable between species. *hda* from *E. coli* failed to complement *pa0947* depletion in *P. aeruginosa* and, when overexpressed, was deleterious in both the depleted and wild-type backgrounds (Fig. 2B). Because RIDA requires productive Hda–DnaA and Hda–β clamp interactions (14, 41, 42), these results suggest that the relevant interaction interfaces are strongly species-dependent. Subtle sequence or conformational differences may impair productive interactions between *E. coli* Hda and *P. aeruginosa* DnaA and the β clamp, thereby compromising efficient inactivation of DnaA after initiation. Thus, even closely related homologs may not function interchangeably across species, underscoring organism-specific adaptations in replication-initiation control. For example, the distantly related *Caulobacter crescentus* encodes its own Hda analog (HdaA) to couple replication initiation to its cell cycle (43), whereas many Gram-positive bacteria lack any Hda homolog and instead use unrelated negative regulators (such as YabA in *Bacillus subtilis*) to achieve once-per-cell-cycle replication control (12, 44). These examples illustrate that replication-initiation control strategies have diverged substantially, emphasizing the need to examine replication regulation in an organism-specific physiological context (45).

To further investigate *pa0947*’s role, we performed a suppressor screen (20, 21, 23) for mutants that could grow in the absence of PA0947 function (Fig. 4). Notably, all four independent suppressors we isolated carried mutations in or near *dnaA* (Fig. 5 and Table S2), providing genetic evidence that PA0947 essentiality is directly tied to restraining DnaA activity. The suppressor mutations clustered in *dnaA* and fell into two functional classes: upstream regulatory indels that reduced *dnaA* expression and C-terminal coding changes expected to compromise DnaA binding to oriC. Biochemical analyses confirmed the expected effects: the upstream regulatory mutations decreased *dnaA* transcription, with *recA* remaining largely unchanged as a control (Fig. 6A and B), and the DnaA variants showed compromised oriC binding (Fig. 6C and D), indicating that suppression converges on dialing down DnaA activity by either lowering its abundance or compromising its productive engagement with oriC.

Mechanistically, our EMSA patterns are consistent with differences in the extent and stability of DnaA–DNA complexes: wt DnaA produced two discrete shifted species on the single DnaA-box-containing probes (Fig. 6C and D and Table 1), which can arise from cooperative DnaA–DnaA interactions and higher-order complex formation (46). In contrast, sup4-DnaA predominantly yielded a single, faster-migrating species with substantial free probe remaining, suggesting reduced complex assembly and/or stability, whereas sup1-DnaA showed markedly impaired binding (Fig. 6C and D). This aligns with DnaA’s domain architecture—domain IV mediates sequence-specific DnaA-box binding, while domain III promotes cooperative multimerization on origin DNA (47). The K490E substitution in sup1-DnaA likely disrupts contacts important for DNA binding, matching the near-absence of shifted species, whereas the +Ile insertion in sup4-DnaA appears to preserve detectable binding but may compromise efficient complex formation (Fig. 6C and D). In line with this biochemical hierarchy, suppression of *pa0947* loss requires a finely tuned reduction in DnaA activity: moderate attenuation (sup2-*dnaA* upstream mutations or the modest binding defect in sup4-DnaA) largely restores near-wt growth and DNA-associated fluorescence profiles, whereas stronger attenuation (sup1-DnaA binding impairment or the pronounced transcriptional downshift in sup3-*dnaA*) permits viability but at a clear fitness cost, with slower growth and greater deviation in DNA-associated fluorescence profiles (Fig. 4 and 6).

Thus, in the absence of PA0947, our data support the idea that fitness is highest within a relatively narrow window of DnaA activity: moderate downshifts are beneficial, whereas excessive compromising can lead to initiation insufficiency and slow growth (19, 48, 49). Notably, in our screen, all suppressors shared mutations in the *dnaA* locus, indicating that bypass of *pa0947*-loss growth defect converges on attenuating DnaA. This pattern parallels *E. coli Δhda* backgrounds, where loss of Hda promotes over-initiation and replication stress, and fitness can be improved when DnaA activity is reduced (19, 48, 50). Conversely, DnaA overproduction is broadly toxic in bacteria because it drives excessive initiation events and consequent DNA damage (34, 51). Hda’s essential role, therefore, is to provide critical control over DnaA-driven initiation, keeping replication initiation within a permissive range compatible with cell viability.

**Fig 7:**
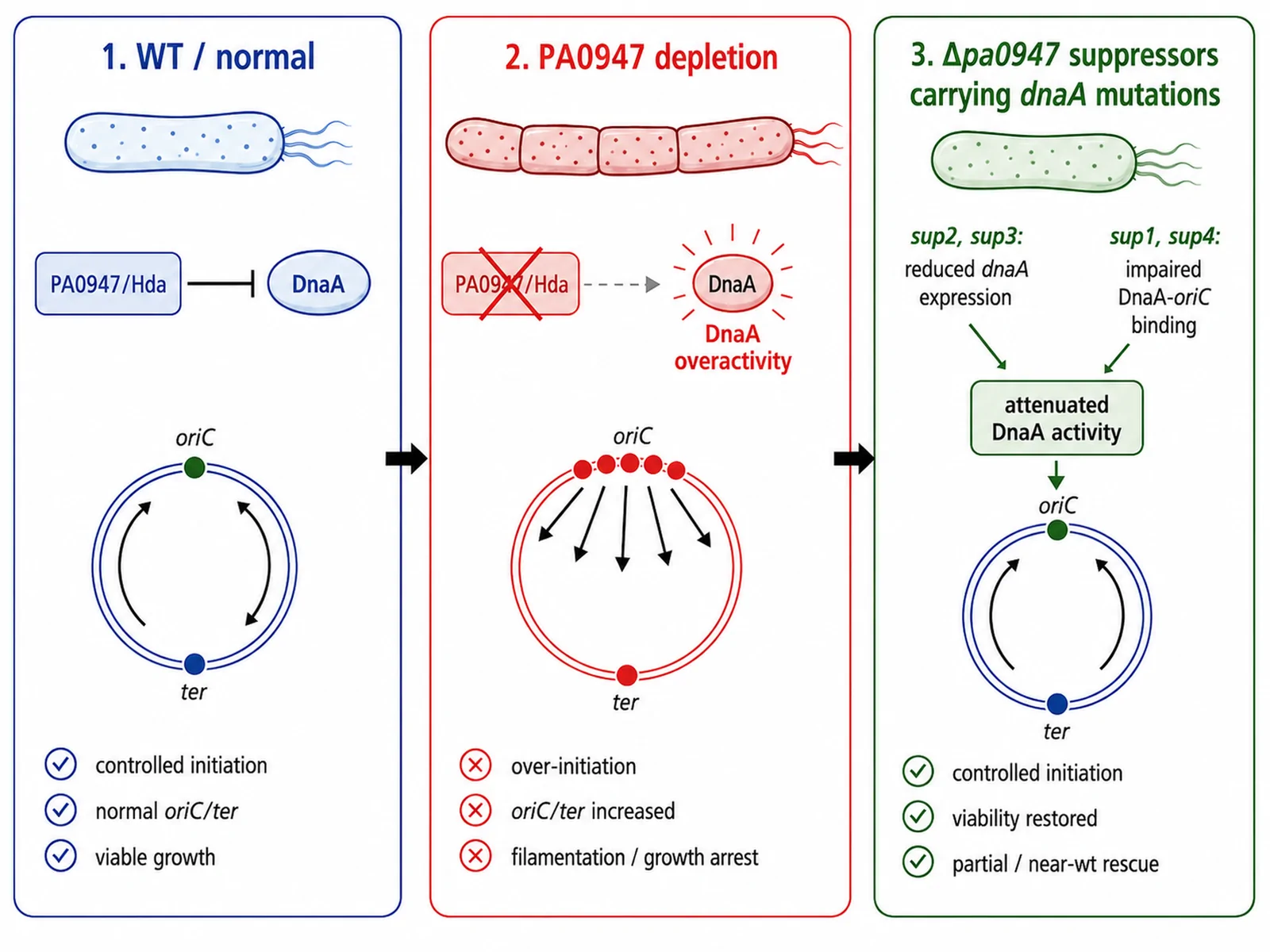
Proposed model for PA0947 function in DnaA-dependent replication-initiation control. Under normal conditions, PA0947/Hda restrains DnaA activity and helps maintain proper replication-initiation control. PA0947 depletion is proposed to cause excessive DnaA-dependent initiation, resulting in increased oriC/ter ratio, filamentation, and growth arrest. *Δpa0947* suppressors carrying *dnaA* mutations bypass PA0947 essentiality by attenuating DnaA activity, either by reducing *dnaA* expression (sup2 and sup3) or by impairing DnaA–oriC binding (sup1 and sup4).

Overall, conditional *pa0947* depletion coupled with suppressor analysis enabled recovery of viable bypass mutants despite *pa0947* essentiality and identified *dnaA* as the convergent bypass locus, linking PA0947 essentiality to tight control of DnaA-dependent initiation within a narrow activity window required for robust initiation control (Fig. 7). More generally, this approach provides a useful framework for dissecting essential yet poorly characterized genes in *P. aeruginosa*.

## MATERIALS AND METHODS

### DNA, plasmids, and bacterial cultures

Details of the oligonucleotides, plasmids, and bacterial strains used in this study are summarized in Table 1. The wild-type *P. aeruginosa* PAO1 strain was obtained from BioSciBio, and all derivative strains were routinely cultured in LB broth (per liter: 10 g tryptone, 10 g NaCl, and 5 g yeast extract) or on LB agar plates prepared by adding 1.5% (wt/vol) agar. Where required, antibiotics were used at the following final concentrations: gentamicin (50 μg/mL) and tetracycline (100 μg/mL). Media were additionally supplemented with selective or inducing agents as needed, including sucrose (10%, wt/vol) and L-arabinose (0.02% or 0.2%, wt/vol). Cultures were incubated at either 30°C or 42°C according to the experimental design. Chemicals were purchased from Sigma-Aldrich or Macklin.

### Plasmid construction

The deletion plasmid (pDel) and temperature-sensitive (ts) rescue plasmid used in this work followed our previously established three-step workflow (20–23): a pDel vector carrying the chromosomal *Δpa0947* allele (Gm^R^, sacB) and a ts rescue plasmid pTS-*pa0947* expressing *pa0947* from its native promoter (Tc^R^, temperature-sensitive replicon) were employed for conditional-growth analyses. Arabinose-inducible plasmids (pOE-*pa0947* and pOE-*ec.hda*) were constructed by placing each coding sequence under P_BAD_ (24) in the broad-host-range vector pBBR1MCS-5 (25). For protein-based assays, wild-type *dnaA* and suppressor alleles were cloned into the pET-28a expression vector to produce His-tagged DnaA proteins. All assemblies were performed using the ClonExpress Ultra One Step Cloning Kit V3 (Vazyme) according to the manufacturer’s instructions, and constructs were sequence-confirmed before use.

### Plasmid-based ts-mutant strain construction

Plasmid-based temperature-sensitive mutant PA0947-ts was generated using our previously established three-step procedure (20–23): a pUC19-derived, nonreplicative (suicide) sacB-based deletion plasmid was first introduced into *P. aeruginosa* PAO1, and single-crossover integrants were selected on gentamicin plates; the ts rescue plasmid carrying *pa0947* under its native promoter was then introduced and selected on tetracycline; finally, sucrose counterselection was applied to resolve the integrated plasmid and obtain the chromosomal *Δpa0947* allele. Candidate *Δpa0947* clones were verified by diagnostic PCR (primer pairs F1/R1 and F2/R2) and Sanger sequencing.

### Spot-plating assay

Spot-plating was used to compare growth phenotypes of the indicated strains. Overnight cultures were normalized to the same optical density (*A*_600_), subjected to 10-fold serial dilutions, and pinned onto LB agar plates containing the indicated supplements or antibiotics using a 48-pin replicator (V&P Scientific). Plates were incubated at 30°C or 42°C as required, and growth was documented after appropriate incubation.

### Fluorescence microscopy

Cell morphology was observed on an Olympus BX53 microscope under phase-contrast optics. For fluorescence staining, cells from the indicated cultures were harvested, fixed with 4% formaldehyde for at least 30 min at room temperature, washed with PBS, and resuspended in PBS. Membranes were stained with Nile red (Sinopharm) at a final concentration of 100 ng/mL for 30 min, followed by washing and resuspension in PBS. Nucleoids were stained with 4′,6-diamidino-2-phenylindole (DAPI; Sinopharm) at a final concentration of 1 μg/mL for 30 min at room temperature, followed by washing and resuspension in PBS before microscopic observation.

### Flow cytometry analysis of DNA-associated fluorescence

Cells were fixed in 70% ethanol for 30 min and washed three times with PBS. Fixed cells were resuspended in PBS to an OD_600_ of 0.6 and stained for 30 min at room temperature with PicoGreen (Solarbio, Beijing, China). The dye stock was diluted 1:200 in dimethyl sulfoxide (DMSO), and 5 μL of this working solution was added to 100 μL of cell suspension. Samples were then diluted with 1 mL PBS (containing PicoGreen at 1:1,000) and analyzed on a CytoFLEX S flow cytometer (Beckman Coulter) equipped with a 488-nm laser. For each sample, ~30,000 events were collected after FSC/SSC gating of the main bacterial population to exclude debris. B525-A fluorescence distributions and median B525-A values were analyzed using CytExpert software (version 2.6; Beckman Coulter).

### Isolation of suppressors

Spontaneous suppressors were selected from the PA0947-ts background as described previously (20, 21, 23). In brief, >1.0 × 10^9^ cells were spread onto LB agar and incubated at a semi-restrictive temperature (40°C) for ~1–2 weeks to allow emergence of suppressor colonies. Candidate colonies were purified by restreaking, retested for growth at 42°C, and verified by diagnostic PCR to confirm retention of the chromosomal *Δpa0947* allele; loss of the ts rescue plasmid was additionally assessed by tetracycline sensitivity at 30°C.

### Genome resequencing analysis

Genome resequencing was carried out for sup1–sup4 and the parental PA0947-ts strain. Genomic DNA was isolated with a commercial kit (TaKaRa), fragmented to ~400 bp using an S2 instrument (Covaris), and used to prepare sequencing libraries with the NEXTflex DNA library kit (Bioo Scientific) following the manufacturer’s instructions. Libraries were sequenced on an Illumina MiSeq (PE300), yielding ~5 million high-quality 300-bp paired-end reads per sample (BioMedical Institute of Shanghai). Reads were mapped to the *P. aeruginosa* PAO1 reference genome (pseudomonas.com) with BWA (52); SNPs and small indels (<50 bp) were called with SAMtools (53), and larger structural variants were assessed using BreakDancer. SNP and indel locations identified in the suppressor mutants are summarized in Table S2.

### RNA extraction and quantitative real-time PCR analysis

Total RNA was isolated from bacterial cultures using the Total RNA Extraction Kit (Solarbio) following the manufacturer’s instructions. cDNA was synthesized from 1 μg of RNA using the PrimeScript RT reagent Kit with gDNA Eraser (TaKaRa) according to the manufacturer’s instructions. *dnaA* transcript levels were quantified by qRT-PCR with two independent primer pairs (dnaA-1 and dnaA-2; amplicons positioned away from the mutation sites) (Table 1), and expression values were normalized to the housekeeping genes *proC* (29). *recA* (30) expression was also measured and normalized to *proC* as a control gene. qRT-PCRs were performed with SYBR Premix Ex Taq II (TaKaRa) on an ABI 7300 system (95°C for 1 min; 40 cycles of 95°C for 15 s, and 60°C for 30 s). Relative expression was calculated by the 2^−ΔΔCt^ method with wt set to 1. Primer sequences are listed in Table 1.

### qPCR-based oriC/ter analysis

Genomic DNA was isolated from the indicated bacterial cultures using a commercial kit (TIANGEN). qPCR was performed with one primer pair targeting the oriC region and one primer pair targeting a chromosomal region approximately opposite oriC, which was used as the ter-proximal reference region. The oriC/ter ratio was calculated from the corresponding Ct values as 2^(Ct_ter − Ct_ori)^. Primer sequences are listed in Table 1.

### Electrophoretic mobility shift assay

Wild-type *dnaA* and suppressor alleles (wt-*dnaA*, sup1-*dnaA,* and sup4-*dnaA*) were cloned into pET-28a to generate His-tagged DnaA proteins. Recombinant proteins were expressed in *E. coli* Rosetta (DE3). Cultures were grown to an OD_600_ of approximately 0.6–0.8, induced with 0.5 mM IPTG, and incubated at 16°C for 20 h. Cells were harvested, resuspended in lysis/binding buffer (50 mM Tris-HCl, pH 8.0, 300 mM NaCl, 10 mM imidazole), disrupted by sonication, and clarified by centrifugation. His-tagged DnaA proteins were purified under native conditions using His-Tag Purification Resin (Roche) and eluted with 250 mM imidazole. Protein preparations were analyzed by Coomassie-stained SDS–PAGE and anti-His western blotting. Two oriC-derived duplex probes (probe 1 and probe 2) were prepared by annealing complementary oligonucleotides (one strand 5′-biotin-labeled). Each probe contains a single high-affinity DnaA box (shown in bold and underlined in Table 1). For specificity control, a mutant version of probe 1 (mut-probe 1, listed in Table 1) carrying a disrupted DnaA-box sequence was also synthesized and used in EMSA. EMSA was performed using the Chemiluminescent EMSA Kit (Beyotime) following the manufacturer’s protocol. Each 20-μL binding reaction contained 1 μg purified DnaA protein and 20 fmol biotin-labeled duplex probe in the manufacturer-supplied EMSA/Gel-Shift binding buffer, which contains optimized poly(dI-dC) as a nonspecific competitor. Samples were incubated at room temperature for 25 min, electrophoresed on 6% native polyacrylamide gels in 0.5 × TBE buffer, and detected by chemiluminescence.

### Statistical analysis

Data are presented as mean ± SD. Statistical significance was evaluated using an unpaired, two-tailed Student’s *t*-test. *P* < 0.05 was considered statistically significant.

## Data Availability

Raw genome-resequencing reads have been deposited in the NCBI Sequence Read Archive (SRA) under BioProject accession PRJNA1511473. All other data are contained within the article and its supporting information.

## SUPPLEMENTAL MATERIAL

Supplemental figures spectrum.00619-26-s0001.docx Figures S1 to S5.

Table S1 spectrum.00619-26-s0002.docx Summary of genetic changes and phenotypes of Δ*pa0947* suppressors.

Table S2 spectrum.00619-26-s0003.docx Small indel and SNP analysis in sup1-4 and Δ*pa0947* (pTS-*pa0947*).

ASM does not own the copyrights to Supplemental Material that may be linked to, or accessed through, an article. The authors have granted ASM a non-exclusive, world-wide license to publish the Supplemental Material files. Please contact the corresponding author directly for reuse.

## References

[1] Davies JC. 2002. *Pseudomonas aeruginosa* in cystic fibrosis: pathogenesis and persistence. Paediatr Respir Rev 3:128–134. doi:10.1016/s1526-0550(02)00003-3 12297059

[2] Elmassry MM, Colmer-Hamood JA, Kopel J, San Francisco MJ, Hamood AN. 2023. Anti-*Pseudomonas aeruginosa* vaccines and therapies: an assessment of clinical trials. Microorganisms 11:916. doi:10.3390/microorganisms11040916 PMC10144840 37110338

[3] Tacconelli E, Carrara E, Savoldi A, Harbarth S, Mendelson M, Monnet DL, Pulcini C, Kahlmeter G, Kluytmans J, Carmeli Y, Ouellette M, Outterson K, Patel J, Cavaleri M, Cox EM, Houchens CR, Grayson ML, Hansen P, Singh N, Theuretzbacher U, Magrini N. 2018. Discovery, research, and development of new antibiotics: the WHO priority list of antibiotic-resistant bacteria and tuberculosis. Lancet Infect Dis 18:318–327. doi:10.1016/S1473-3099(17)30753-3 29276051

[4] Juhas M, Eberl L, Church GM. 2012. Essential genes as antimicrobial targets and cornerstones of synthetic biology. Trends Biotechnol 30:601–607. doi:10.1016/j.tibtech.2012.08.002 22951051

[5] Fields FR, Lee SW, McConnell MJ. 2017. Using bacterial genomes and essential genes for the development of new antibiotics. Biochem Pharmacol 134:74–86. doi:10.1016/j.bcp.2016.12.002 PMC5411290 27940263

[6] Ward RD, Tran JS, Banta AB, Bacon EE, Rose WE, Peters JM. 2024. Essential gene knockdowns reveal genetic vulnerabilities and antibiotic sensitivities in *Acinetobacter baumannii*. mBio 15:e0205123. doi:10.1128/mbio.02051-23 PMC10865783 38126769

[7] Lee SA, Gallagher LA, Thongdee M, Staudinger BJ, Lippman S, Singh PK, Manoil C. 2015. General and condition-specific essential functions of *Pseudomonas aeruginosa*. Proc Natl Acad Sci USA 112:5189–5194. doi:10.1073/pnas.1422186112 PMC4413342 25848053

[8] Naveed M, Chaudhry Z, Ali Z, Amjad M, Zulfiqar F, Numan A. 2018. Annotation and curation of hypothetical proteins: prioritizing targets for experimental study. Adv Life Sci 5:73–87.

[9] Chalker AF, Lunsford RD. 2002. Rational identification of new antibacterial drug targets that are essential for viability using a genomics-based approach. Pharmacology & Therapeutics 95:1–20. doi:10.1016/S0163-7258(02)00222-X 12163125

[10] Rost B. 1999. Twilight zone of protein sequence alignments. Protein Eng 12:85–94. doi:10.1093/protein/12.2.85 10195279

[11] Kato J, Katayama T. 2001. Hda, a novel DnaA-related protein, regulates the replication cycle in *Escherichia coli*. EMBO J 20:4253–4262. doi:10.1093/emboj/20.15.4253 PMC149159 11483528

[12] Kurz M, Dalrymple B, Wijffels G, Kongsuwan K. 2004. Interaction of the sliding clamp beta-subunit and Hda, a DnaA-related protein. J Bacteriol 186:3508–3515. doi:10.1128/JB.186.11.3508-3515.2004 PMC415757 15150238

[13] Nakamura K, Katayama T. 2010. Novel essential residues of Hda for interaction with DnaA in the regulatory inactivation of DnaA: unique roles for Hda AAA Box VI and VII motifs. Mol Microbiol 76:302–317. doi:10.1111/j.1365-2958.2010.07074.x 20132442

[14] Su’etsugu M, Nakamura K, Keyamura K, Kudo Y, Katayama T. 2008. Hda monomerization by ADP binding promotes replicase clamp-mediated DnaA-ATP hydrolysis. J Biol Chem 283:36118–36131. doi:10.1074/jbc.M803158200 PMC2662290 18977760

[15] Camara JE, Breier AM, Brendler T, Austin S, Cozzarelli NR, Crooke E. 2005. Hda inactivation of DnaA is the predominant mechanism preventing hyperinitiation of *Escherichia coli* DNA replication. EMBO Rep 6:736–741. doi:10.1038/sj.embor.7400467 PMC1369143 16041320

[16] Knöppel A, Broström O, Gras K, Elf J, Fange D. 2023. Regulatory elements coordinating initiation of chromosome replication to the *Escherichia coli* cell cycle. Proc Natl Acad Sci USA 120:e2213795120. doi:10.1073/pnas.2213795120 PMC10235992 37220276

[17] Zeng D, Zhao J, Chung HS, Guan Z, Raetz CRH, Zhou P. 2013. Mutants resistant to LpxC inhibitors by rebalancing cellular homeostasis. J Biol Chem 288:5475–5486. doi:10.1074/jbc.M112.447607 PMC3581379 23316051

[18] van Leeuwen J, Pons C, Tan G, Wang ZY, Hou J, Weile J, Gebbia M, Liang W, Shuteriqi E, Li Z, Lopes M, Ušaj M, Dos Santos Lopes A, van Lieshout N, Myers CL, Roth FP, Aloy P, Andrews BJ, Boone C. 2020. Systematic analysis of bypass suppression of essential genes. Mol Syst Biol 16:e9828. doi:10.15252/msb.20209828 PMC7507402 32939983

[19] Charbon G, Riber L, Cohen M, Skovgaard O, Fujimitsu K, Katayama T, Løbner-Olesen A. 2011. Suppressors of DnaA(ATP) imposed overinitiation in *Escherichia coli*. Mol Microbiol 79:914–928. doi:10.1111/j.1365-2958.2010.07493.x 21299647

[20] Yang Z, Zhang Z, Zhu J, Ma Y, Wang J, Liu J. 2022. Analysis of the plasmid-based *ts* Allele of *PA0006* reveals its function in regulation of cell morphology and biosynthesis of core lipopolysaccharide in *Pseudomonas aeruginosa*. Appl Environ Microbiol 88:e0048022. doi:10.1128/aem.00480-22 PMC9317947 35762790

[21] Tian L, Yang Z, Wang J, Liu J. 2023. Analysis of the plasmid-based *ts*-mutant Δ*fabA/pTS-fabA* reveals its lethality under aerobic growth conditions that is suppressed by mild overexpression of *desA* at a restrictive temperature in *Pseudomonas aeruginosa*. Microbiol Spectr 11:e0133823. doi:10.1128/spectrum.01338-23 PMC10269440 37191499

[22] Zhu J, Zhao H, Yang Z. 2024. Genetic analysis of the ts-lethal mutant Δpa0665/pTS-pa0665 reveals its role in cell morphology and oxidative phosphorylation in *Pseudomonas aeruginosa*. Genes (Basel) 15:590. doi:10.3390/genes15050590 PMC11120684 38790219

[23] Chen Z, Lu J, Tang Q, Yang Z. 2025. Conditional lethality and suppressor analysis of plasmid-based temperature-sensitive fabZ expression in *Pseudomonas aeruginosa*. J Biol Chem 301:108553. doi:10.1016/j.jbc.2025.108553 PMC12152623 40294648

[24] Guzman LM, Belin D, Carson MJ, Beckwith J. 1995. Tight regulation, modulation, and high-level expression by vectors containing the arabinose PBAD promoter. J Bacteriol 177:4121–4130. doi:10.1128/jb.177.14.4121-4130.1995 PMC177145 7608087

[25] Kovach ME, Elzer PH, Hill DS, Robertson GT, Farris MA, Roop RM 2nd, Peterson KM. 1995. Four new derivatives of the broad-host-range cloning vector pBBR1MCS, carrying different antibiotic-resistance cassettes. Gene 166:175–176. doi:10.1016/0378-1119(95)00584-1 8529885

[26] Singer VL, Jones LJ, Yue ST, Haugland RP. 1997. Characterization of PicoGreen reagent and development of a fluorescence-based solution assay for double-stranded DNA quantitation. Anal Biochem 249:228–238. doi:10.1006/abio.1997.2177 9212875

[27] Dragan AI, Casas-Finet JR, Bishop ES, Strouse RJ, Schenerman MA, Geddes CD. 2010. Characterization of PicoGreen interaction with dsDNA and the origin of its fluorescence enhancement upon binding. Biophys J 99:3010–3019. doi:10.1016/j.bpj.2010.09.012 PMC2965993 21044599

[28] Zhao X, Jin Y, Bai F, Cheng Z, Wu W, Pan X. 2022. *Pseudomonas aeruginosa* phosphate transporter PitA (PA4292) controls susceptibility to aminoglycoside antibiotics by regulating the proton motive force. Antimicrob Agents Chemother 66:e00992–22. doi:10.1128/aac.00992-22 PMC9765264 36346250

[29] Savli H, Karadenizli A, Kolayli F, Gundes S, Ozbek U, Vahaboglu H. 2003. Expression stability of six housekeeping genes: a proposal for resistance gene quantification studies of *Pseudomonas aeruginosa* by real-time quantitative RT-PCR. J Med Microbiol 52:403–408. doi:10.1099/jmm.0.05132-0 12721316

[30] Meng L, Cao X, Li C, Li J, Xie H, Shi J, Han M, Shen H, Liu C. 2023. Housekeeping gene stability in *Pesudomonas aeruginosa* PAO1 under the pressure of commonly used antibiotics in molecular microbiology assays. Front Microbiol 14:1140515. doi:10.3389/fmicb.2023.1140515 PMC10040570 36992935

[31] Yee TW, Smith DW. 1990. Pseudomonas chromosomal replication origins: a bacterial class distinct from *Escherichia coli*-type origins. Proc Natl Acad Sci USA 87:1278–1282. doi:10.1073/pnas.87.4.1278 PMC53457 2106132

[32] Stover CK, Pham XQ, Erwin AL, Mizoguchi SD, Warrener P, Hickey MJ, Brinkman FS, Hufnagle WO, Kowalik DJ, Lagrou M, et al.. 2000. Complete genome sequence of *Pseudomonas aeruginosa* PAO1, an opportunistic pathogen. Nature 406:959–964. doi:10.1038/35023079 10984043

[33] Fujimitsu K, Su’etsugu M, Yamaguchi Y, Mazda K, Fu N, Kawakami H, Katayama T. 2008. Modes of overinitiation, dnaA gene expression, and inhibition of cell division in a novel cold-sensitive hda mutant of *Escherichia coli**.* J Bacteriol 190:5368–5381. doi:10.1128/JB.00044-08 PMC2493284 18502852

[34] Charbon G, Bjørn L, Mendoza-Chamizo B, Frimodt-Møller J, Løbner-Olesen A. 2014. Oxidative DNA damage is instrumental in hyperreplication stress-induced inviability of *Escherichia coli*. Nucleic Acids Res 42:13228–13241. doi:10.1093/nar/gku1149 PMC4245963 25389264

[35] Su’etsugu M, Takata M, Kubota T, Matsuda Y, Katayama T. 2004. Molecular mechanism of DNA replication-coupled inactivation of the initiator protein in *Escherichia coli*: interaction of DnaA with the sliding clamp-loaded DNA and the sliding clamp-Hda complex. Genes Cells 9:509–522. doi:10.1111/j.1356-9597.2004.00741.x 15189445

[36] Katayama T, Ozaki S, Keyamura K, Fujimitsu K. 2010. Regulation of the replication cycle: conserved and diverse regulatory systems for DnaA and oriC. Nat Rev Microbiol 8:163–170. doi:10.1038/nrmicro2314 20157337

[37] Zakrzewska-Czerwińska J, Jakimowicz D, Zawilak-Pawlik A, Messer W. 2007. Regulation of the initiation of chromosomal replication in bacteria. FEMS Microbiol Rev 31:378–387. doi:10.1111/j.1574-6976.2007.00070.x 17459114

[38] Camara JE, Skarstad K, Crooke E. 2003. Controlled initiation of chromosomal replication in *Escherichia coli* requires functional Hda protein. J Bacteriol 185:3244–3248. doi:10.1128/JB.185.10.3244-3248.2003 PMC154069 12730188

[39] Nievera C, Torgue JJ-C, Grimwade JE, Leonard AC. 2006. SeqA blocking of DnaA-oriC interactions ensures staged assembly of the *E. coli* pre-RC. Mol Cell 24:581–592. doi:10.1016/j.molcel.2006.09.016 PMC1939805 17114060

[40] Brézellec P, Hoebeke M, Hiet M-S, Pasek S, Ferat J-L. 2006. DomainSieve: a protein domain-based screen that led to the identification of dam-associated genes with potential link to DNA maintenance. Bioinformatics 22:1935–1941. doi:10.1093/bioinformatics/btl336 16787973

[41] Su’etsugu M, Harada Y, Keyamura K, Matsunaga C, Kasho K, Abe Y, Ueda T, Katayama T. 2013. The DnaA N-terminal domain interacts with Hda to facilitate replicase clamp-mediated inactivation of DnaA. Environ Microbiol 15:3183–3195. doi:10.1111/1462-2920.12147 23679057

[42] Su’etsugu M, Shimuta T-R, Ishida T, Kawakami H, Katayama T. 2005. Protein associations in DnaA-ATP hydrolysis mediated by the Hda-replicase clamp complex. J Biol Chem 280:6528–6536. doi:10.1074/jbc.M412060200 15611053

[43] Collier J, Shapiro L. 2009. Feedback control of DnaA-mediated replication initiation by replisome-associated HdaA protein in *Caulobacter*. J Bacteriol 191:5706–5716. doi:10.1128/JB.00525-09 PMC2737981 19633089

[44] Goranov AI, Breier AM, Merrikh H, Grossman AD. 2009. YabA of *Bacillus subtilis* controls DnaA-mediated replication initiation but not the transcriptional response to replication stress. Mol Microbiol 74:454–466. doi:10.1111/j.1365-2958.2009.06876.x PMC2823125 19737352

[45] Leonard AC, Grimwade JE. 2015. The orisome: structure and function. Front Microbiol 6:545. doi:10.3389/fmicb.2015.00545 PMC4451416 26082765

[46] Miller DT, Grimwade JE, Betteridge T, Rozgaja T, Torgue JJ-C, Leonard AC. 2009. Bacterial origin recognition complexes direct assembly of higher-order DnaA oligomeric structures. Proc Natl Acad Sci USA 106:18479–18484. doi:10.1073/pnas.0909472106 PMC2773971 19833870

[47] Fujikawa N, Kurumizaka H, Nureki O, Terada T, Shirouzu M, Katayama T, Yokoyama S. 2003. Structural basis of replication origin recognition by the DnaA protein. Nucleic Acids Res 31:2077–2086. doi:10.1093/nar/gkg309 PMC153737 12682358

[48] Riber L, Olsson JA, Jensen RB, Skovgaard O, Dasgupta S, Marinus MG, Løbner-Olesen A. 2006. Hda-mediated inactivation of the DnaA protein and dnaA gene autoregulation act in concert to ensure homeostatic maintenance of the *Escherichia coli* chromosome. Genes Dev 20:2121–2134. doi:10.1101/gad.379506 PMC1536062 16882985

[49] Morigen Løbner-Olesen A, Skarstad K. 2003. Titration of the *Escherichia coli* DnaA protein to excess datA sites causes destabilization of replication forks, delayed replication initiation and delayed cell division. Mol Microbiol 50:349–362. doi:10.1046/j.1365-2958.2003.03695.x 14507385

[50] Charbon G, Campion C, Chan SHJ, Bjørn L, Weimann A, da Silva LCN, Jensen PR, Løbner-Olesen A. 2017. Re-wiring of energy metabolism promotes viability during hyperreplication stress in *E. coli*. PLoS Genet 13:e1006590. doi:10.1371/journal.pgen.1006590 PMC5302844 28129339

[51] Simmons LA, Breier AM, Cozzarelli NR, Kaguni JM. 2004. Hyperinitiation of DNA replication in *Escherichia coli* leads to replication fork collapse and inviability. Mol Microbiol 51:349–358. doi:10.1046/j.1365-2958.2003.03842.x 14756777

[52] Li H., Durbin R. 2009. Fast and accurate short read alignment with Burrows-Wheeler transform. Bioinformatics 25:1754–1760. doi:10.1093/bioinformatics/btp324 PMC2705234 19451168

[53] Li H, Handsaker B, Wysoker A, Fennell T, Ruan J, Homer N, Marth G, Abecasis G, Durbin R. 2009. The sequence alignment/map format and SAMtools. Bioinformatics 25:2078–2079. doi:10.1093/bioinformatics/btp352 PMC2723002 19505943

