# Supplementary material for: Analysis of the plasmid-based ts-mutant Δpa0947 (pTS-pa0947) reveals pa0947 essentiality and dnaA-centered suppressor bypass in Pseudomonas aeruginosa
Source: Microbiol Spectr. 2026 Sep 22;14(11):e00619-26. doi: 10.1128/spectrum.00619-26 (PMC13729189; doi:10.1128/spectrum.00619-26)
Supplement: Supplementary file 1 [file spectrum.00619-26-s0001.docx]

**Supplementary Figure S1. Median B525-A fluorescence from PicoGreen-stained and unstained cells.**

Median B525-A values from wt and *Δpa0947*(pTS-*pa0947*) cells grown at 30°C or shifted to 42°C, with or without PicoGreen staining. Data are mean ± SD (*n* = 3). ***, *p* < 0.001 versus wt at 42°C.

**Supplementary Figure S2. FSC-A profiles and oriC/ter ratios of suppressor strains.**

***A and B,*** Flow-cytometric overlays of FSC-A and median B525-A values for wt and suppressor strains grown in LB medium at 30°C. **, *p* < 0.01; ***, *p* < 0.001 versus wt. ***C,*** qPCR-based oriC/ter analysis of wt and suppressor strains grown at 30°C. Data are mean ± SD (*n* = 3). *, *p* < 0.05.

**Supplementary Figure S3. *dnaA* transcript levels in wt and sup1–sup4 measured by qRT–PCR with the dnaA-2 primer set and normalized to *proC*.** Data are mean ± SD (*n* = 3). **, *p* < 0.01; ***, *p* < 0.001 (vs wt).

**Supplementary Figure S4. Expression, purification, and verification of His-tagged DnaA variants used for EMSA.**

***A,*** Coomassie brilliant blue-stained SDS–PAGE analysis of wt-DnaA, sup1-DnaA, and sup4-DnaA expression and purification. Lane M, protein marker; lanes 1–3, wt-DnaA samples before IPTG induction, after IPTG induction, and after Ni-affinity purification, respectively; lanes 4–6, sup1-DnaA samples analyzed in the same order; lanes 7–9, sup4-DnaA samples analyzed in the same order. ***B,*** Anti-His western blotting of the purified His-tagged wt-DnaA, sup1-DnaA, and sup4-DnaA preparations. The position of His-DnaA is indicated.

**Supplementary Figure S5. Control EMSA showing specific DnaA binding to the DnaA box in probe 1.** EMSA of purified His-tagged wt DnaA with wild-type probe 1 or mut-probe 1 containing a mutated DnaA-box sequence (Table 1).
