# Supplementary material for: Analysis of the plasmid-based ts-mutant Δpa0947 (pTS-pa0947) reveals pa0947 essentiality and dnaA-centered suppressor bypass in Pseudomonas aeruginosa
Source: Microbiol Spectr. 2026 Sep 22;14(11):e00619-26. doi: 10.1128/spectrum.00619-26 (PMC13729189; doi:10.1128/spectrum.00619-26)
Supplement: Supplementary file 2 [file spectrum.00619-26-s0002.docx]

**Table S1. Summary of genetic changes and phenotypes of *Δpa0947* suppressors.**

| **Suppressor** | **Genetic changes** | **Growth/morphology** | **PicoGreen-associated fluorescence** | ***dnaA* expression** | **DnaA–oriC binding** |
| --- | --- | --- | --- | --- | --- |
| sup1 | dnaA coding mutation, c.1468A>G, p.Lys490Glu | Slow growth; filamentous/chain-like cells | Broad/right-shifted profile | Near wt | Strongly impaired |
| sup2 | dnaA upstream −9_−8insTTGGG | Near-wt growth and morphology | Modest right shift | Reduced to ~25% of wt | N/A |
| sup3 | dnaA upstream −62_−54delCCGTCCGGC; PA4292 nonsense mutation | Slow growth; mostly short rods | Left-shifted profile | Reduced to <5% of wt | N/A |
| sup4 | dnaA in-frame ATC insertion, +Ile | Near-wt growth and morphology | Near-wt profile | Near wt | Reduced/altered |
