# Supplementary material for: Analysis of the plasmid-based ts-mutant Δpa0947 (pTS-pa0947) reveals pa0947 essentiality and dnaA-centered suppressor bypass in Pseudomonas aeruginosa
Source: Microbiol Spectr. 2026 Sep 22;14(11):e00619-26. doi: 10.1128/spectrum.00619-26 (PMC13729189; doi:10.1128/spectrum.00619-26)
Supplement: Supplementary file 3 [file spectrum.00619-26-s0003.docx]

| **Supplementary table S2. Small indel and SNP** **analysis in sup1-4 and *Δpa0947*(pTS-*pa0947*)** | | | | | | | | | | | | | | | | |  |
| --- | --- | --- | --- | --- | --- | --- | --- | --- | --- | --- | --- | --- | --- | --- | --- | --- | --- |
|  |  |  |  |  |  |  |  |  |  |  |  |  |  |  |  |  |  |
| **(A) Indel loci specific to sup1-4, not present in *Δpa0947*(pTS-*pa0947*)** | | | | | | | | | | | | | | | | |  |
| **sup1** | | | | | | | | | ***Δpa0947*(pTS-*pa0947*)** | | | | | | | |  |
| **POS** | **REF** | **ALT** | **alt-coverage** | **all-coverage** | **alt-precent** | **Gene_loci** | **Gene_ID** | **function** | **POS** | **REF** | **ALT** | **alt-coverage** | **all-coverage** | **alt-precent** | **Gene_loci** | **Gene_ID** | **function** |
| None detected. | | | | | | | | | NA |  |  |  |  |  |  |  |  |
|  |  |  |  |  |  |  |  |  |  |  |  |  |  |  |  |  |  |
| **sup2** | | | | | | | | | ***Δpa0947*(pTS-*pa0947*)** | | | | | | | |  |
| **POS** | **REF** | **ALT** | **alt-coverage** | **all-coverage** | **alt-precent** | **Gene_loci** | **Gene_ID** | **function** | **POS** | **REF** | **ALT** | **alt-coverage** | **all-coverage** | **alt-precent** | **Gene_loci** | **Gene_ID** |  |
| 474 | G | GTTGGG | 308 | 308 | 1 |  |  |  | NA |  |  |  |  |  |  |  |  |
|  |  |  |  |  |  |  |  |  |  |  |  |  |  |  |  |  |  |
| **sup3** | | | | | | | | | ***Δpa0947*(pTS-*pa0947*)** | | | | | | | |  |
| **POS** | **REF** | **ALT** | **alt-coverage** | **all-coverage** | **alt-precent** | **Gene_loci** | **Gene_ID** | **function** | **POS** | **REF** | **ALT** | **alt-coverage** | **all-coverage** | **alt-precent** | **Gene_loci** | **Gene_ID** |  |
| 420 | CCCGTCCGGC | C | 315 | 315 | 1 |  |  |  | NA |  |  |  |  |  |  |  |  |
| 4816257 | GCAGGGCATGACCTACGCC | G | 103 | 103 | 1 | 1215 | PA4292 | probable phosphate transporter | NA |  |  |  |  |  |  |  |  |
|  |  |  |  |  |  |  |  |  |  |  |  |  |  |  |  |  |  |
| **sup4** | | | | | | | | | ***Δpa0947*(pTS-*pa0947*)** | | | | | | | |  |
| **POS** | **REF** | **ALT** | **alt-coverage** | **all-coverage** | **alt-precent** | **Gene_loci** | **Gene_ID** | **function** | **POS** | **REF** | **ALT** | **alt-coverage** | **all-coverage** | **alt-precent** | **Gene_loci** | **Gene_ID** |  |
| 1598 | C | CATC | 206 | 206 | 1 | 1116 | PA0001 | chromosomal replication initiator protein DnaA | NA |  |  |  |  |  |  |  |  |
|  |  |  |  |  |  |  |  |  |  |  |  |  |  |  |  |  |  |
| **(B) SNP loci specific to sup1-4, not present in *Δpa0947*(pTS-*pa0947*)** | | | | | | | | | | | | | | | | |  |
| **sup1** | | | | | | | | | ***Δpa0947*(pTS-*pa0947*)** | | | | | | | |  |
| **POS** | **REF** | **ALT** | **alt-coverage** | **all-coverage** | **alt-precent** | **Gene_loci** | **Gene_ID** | **function** | **POS** | **REF** | **ALT** | **alt-coverage** | **all-coverage** | **alt-precent** | **Gene_loci** | **Gene_ID** | **function** |
| 1950 | A | G | 158 | 161 | 0.981366 | 1468 | PA0001 | chromosomal replication initiator protein DnaA | NA |  |  |  |  |  |  |  |  |
|  |  |  |  |  |  |  |  |  |  |  |  |  |  |  |  |  |  |
| **sup2** | | | | | | | | | ***Δpa0947*(pTS-*pa0947*)** | | | | | | | |  |
| **POS** | **REF** | **ALT** | **alt-coverage** | **all-coverage** | **alt-precent** | **Gene_loci** | **Gene_ID** | **function** | **POS** | **REF** | **ALT** | **alt-coverage** | **all-coverage** | **alt-precent** | **Gene_loci** | **Gene_ID** |  |
| None detected. | | | | | | | | | NA |  |  |  |  |  |  |  |  |
|  |  |  |  |  |  |  |  |  |  |  |  |  |  |  |  |  |  |
| **sup3** | | | | | | | | | ***Δpa0947*(pTS-*pa0947*)** | | | | | | | |  |
| **POS** | **REF** | **ALT** | **alt-coverage** | **all-coverage** | **alt-precent** | **Gene_loci** | **Gene_ID** | **function** | **POS** | **REF** | **ALT** | **alt-coverage** | **all-coverage** | **alt-precent** | **Gene_loci** | **Gene_ID** |  |
| None detected. | | | | | | | | | NA |  |  |  |  |  |  |  |  |
|  |  |  |  |  |  |  |  |  |  |  |  |  |  |  |  |  |  |
| **sup4** | | | | | | | | | ***Δpa0947*(pTS-*pa0947*)** | | | | | | | |  |
| **POS** | **REF** | **ALT** | **alt-coverage** | **all-coverage** | **alt-precent** | **Gene_loci** | **Gene_ID** | **function** | **POS** | **REF** | **ALT** | **alt-coverage** | **all-coverage** | **alt-precent** | **Gene_loci** | **Gene_ID** |  |
| None detected. | | | | | | | | | NA |  |  |  |  |  |  |  |  |
|  |  |  |  |  |  |  |  |  |  |  |  |  |  |  |  |  |  |
| **(C) Indel loci in both sup1-4 and *Δpa0947*(pTS-*pa0947*)** | | | | | | | | | | | | | | | | |  |
| **sup1** | | | | | | | | | ***Δpa0947*(pTS-*pa0947*)** | | | | | | | |  |
| **POS** | **REF** | **ALT** | **alt-coverage** | **all-coverage** | **alt-precent** | **Gene_loci** | **Gene_ID** | **function** | **POS** | **REF** | **ALT** | **alt-coverage** | **all-coverage** | **alt-precent** | **Gene_loci** | **Gene_ID** | **function** |
| 169283 | CG | C | 126 | 126 | 1 |  |  |  | 169283 | CG | C | 98 | 98 | 1 |  |  |  |
| 411125 | AC | A | 104 | 104 | 1 |  |  |  | 411125 | AC | A | 104 | 104 | 1 |  |  |  |
| 667028 | G | GC | 125 | 125 | 1 |  |  |  | 667028 | G | GC | 104 | 104 | 1 |  |  |  |
| 740419 | G | GC | 82 | 82 | 1 | 217 | PA0683 | probable type II secretion system protein | 740419 | G | GC | 90 | 90 | 1 | 217 | PA0683 | probable type II secretion system protein |
| 816529 | G | GC | 80 | 80 | 1 |  |  |  | 816529 | G | GC | 102 | 102 | 1 |  |  |  |
| 891099 | A | AC | 108 | 108 | 1 |  |  |  | 891099 | A | AC | 91 | 91 | 1 |  |  |  |
| 1116213 | G | GC | 95 | 95 | 1 | 1 | PA1029 | hypothetical protein | 1116213 | G | GC | 104 | 104 | 1 | 1 | PA1029 | hypothetical protein |
| 1215657 | A | AG | 65 | 65 | 1 | 374 | PA1122 | probable peptide deformylase | 1215657 | A | AG | 97 | 97 | 1 | 374 | PA1122 | probable peptide deformylase |
| 1275766 | GA | G | 84 | 84 | 1 | 33 | PA1174 | periplasmic nitrate reductase protein NapA | 1275766 | GA | G | 105 | 105 | 1 | 33 | PA1174 | periplasmic nitrate reductase protein NapA |
| 1440622 | CA | C | 72 | 72 | 1 | 1917 | PA1327 | probable protease | 1440622 | CA | C | 85 | 85 | 1 | 1917 | PA1327 | probable protease |
| 1445357 | A | AG | 82 | 82 | 1 |  |  |  | 1445357 | A | AG | 65 | 65 | 1 |  |  |  |
| 1467482 | A | AG | 103 | 103 | 1 |  |  |  | 1467482 | A | AG | 96 | 96 | 1 |  |  |  |
| 1467483 | C | CG | 102 | 102 | 1 |  |  |  | 1467483 | C | CG | 98 | 98 | 1 |  |  |  |
| 1835045 | G | GC | 79 | 79 | 1 | 653 | PA1685 | enolase-phosphatase E-1 | 1835045 | G | GC | 76 | 76 | 1 | 653 | PA1685 | enolase-phosphatase E-1 |
| 2169348 | A | AG | 52 | 52 | 1 |  |  |  | 2169348 | A | AG | 72 | 72 | 1 |  |  |  |
| 2186927 | G | GC | 48 | 48 | 1 |  |  |  | 2186927 | G | GC | 62 | 62 | 1 |  |  |  |
| 2195457 | G | GC | 81 | 81 | 1 |  |  |  | 2195457 | G | GC | 73 | 73 | 1 |  |  |  |
| 2239555 | A | AG | 63 | 63 | 1 |  |  |  | 2239555 | A | AG | 52 | 52 | 1 |  |  |  |
| 2342110 | C | CT | 88 | 88 | 1 |  |  |  | 2342110 | C | CT | 114 | 114 | 1 |  |  |  |
| 2355771 | A | AG | 56 | 56 | 1 | 88 | PA2139 | hypothetical protein | 2355771 | A | AG | 57 | 57 | 1 | 88 | PA2139 | hypothetical protein |
| 2356681 | GC | G | 70 | 70 | 1 | 514 | PA2141 | hypothetical protein | 2356681 | GC | G | 97 | 97 | 1 | 514 | PA2141 | hypothetical protein |
| 2532046 | G | GC | 49 | 49 | 1 |  |  |  | 2532046 | G | GC | 75 | 75 | 1 |  |  |  |
| 2753522 | G | GC | 52 | 52 | 1 | 924 | PA2452 | hypothetical protein | 2753522 | G | GC | 72 | 72 | 1 | 924 | PA2452 | hypothetical protein |
| 2807693 | TCGGCCAGC | T | 65 | 65 | 1 | 225 | PA2492 | transcriptional regulator MexT | 2807693 | TCGGCCAGC | T | 80 | 80 | 1 | 225 | PA2492 | transcriptional regulator MexT |
| 3016844 | G | GC | 71 | 71 | 1 | 41 | PA2668 | hypothetical protein | 3016844 | G | GC | 88 | 88 | 1 | 41 | PA2668 | hypothetical protein |
| 3083196 | A | AG | 82 | 82 | 1 | 3347 | PA2727 | hypothetical protein | 3083196 | A | AG | 77 | 77 | 1 | 3347 | PA2727 | hypothetical protein |
| 3919508 | G | GC | 97 | 97 | 1 |  |  |  | 3919508 | G | GC | 76 | 76 | 1 |  |  |  |
| 4539468 | G | GC | 106 | 106 | 1 | 313 | PA4059 | hypothetical protein | 4539468 | G | GC | 82 | 82 | 1 | 313 | PA4059 | hypothetical protein |
| 4888194 | A | AG | 91 | 91 | 1 | 2 | PA4360 | hypothetical protein | 4888194 | A | AG | 111 | 111 | 1 | 2 | PA4360 | hypothetical protein |
| 5033101 | G | GC | 97 | 97 | 1 | 1614 | PA4496 | probable binding protein component of ABC transporter | 5033101 | G | GC | 97 | 98 | 0.989796 | 1614 | PA4496 | probable binding protein component of ABC transporter |
| 5071543 | AACTG | A | 115 | 117 | 0.982906 |  |  |  | 5071543 | AACTG | A | 118 | 118 | 1 |  |  |  |
| 5472415 | C | CG | 106 | 106 | 1 |  |  |  | 5472415 | C | CG | 102 | 102 | 1 |  |  |  |
| 5655220 | C | CCGG | 115 | 116 | 0.991379 | 410 | PA5024 | conserved hypothetical protein | 5655220 | C | CCGG | 81 | 83 | 0.975904 | 410 | PA5024 | conserved hypothetical protein |
|  |  |  |  |  |  |  |  |  |  |  |  |  |  |  |  |  |  |
| **sup2** | | | | | | | | | ***Δpa0947*(pTS-*pa0947*)** | | | | | | | |  |
| **POS** | **REF** | **ALT** | **alt-coverage** | **all-coverage** | **alt-precent** | **Gene_loci** | **Gene_ID** | **function** | **POS** | **REF** | **ALT** | **alt-coverage** | **all-coverage** | **alt-precent** | **Gene_loci** | **Gene_ID** |  |
| 169283 | CG | C | 181 | 181 | 1 |  |  |  | 169283 | CG | C | 98 | 98 | 1 |  |  |  |
| 411125 | AC | A | 147 | 147 | 1 |  |  |  | 411125 | AC | A | 104 | 104 | 1 |  |  |  |
| 667028 | G | GC | 126 | 126 | 1 |  |  |  | 667028 | G | GC | 104 | 104 | 1 |  |  |  |
| 740419 | G | GC | 185 | 185 | 1 | 217 | PA0683 | probable type II secretion system protein | 740419 | G | GC | 90 | 90 | 1 | 217 | PA0683 |  |
| 816529 | G | GC | 175 | 175 | 1 |  |  |  | 816529 | G | GC | 102 | 102 | 1 |  |  |  |
| 891099 | A | AC | 142 | 142 | 1 |  |  |  | 891099 | A | AC | 91 | 91 | 1 |  |  |  |
| 1116213 | G | GC | 208 | 208 | 1 | 1 | PA1029 | hypothetical protein | 1116213 | G | GC | 104 | 104 | 1 | 1 | PA1029 |  |
| 1215657 | A | AG | 173 | 173 | 1 | 374 | PA1122 | probable peptide deformylase | 1215657 | A | AG | 97 | 97 | 1 | 374 | PA1122 |  |
| 1275766 | GA | G | 151 | 151 | 1 | 33 | PA1174 | periplasmic nitrate reductase protein NapA | 1275766 | GA | G | 105 | 105 | 1 | 33 | PA1174 |  |
| 1440622 | CA | C | 159 | 159 | 1 | 1917 | PA1327 | probable protease | 1440622 | CA | C | 85 | 85 | 1 | 1917 | PA1327 |  |
| 1445357 | A | AG | 171 | 171 | 1 |  |  |  | 1445357 | A | AG | 65 | 65 | 1 |  |  |  |
| 1467482 | A | AG | 160 | 160 | 1 |  |  |  | 1467482 | A | AG | 96 | 96 | 1 |  |  |  |
| 1467483 | C | CG | 163 | 163 | 1 |  |  |  | 1467483 | C | CG | 98 | 98 | 1 |  |  |  |
| 1835045 | G | GC | 179 | 179 | 1 | 653 | PA1685 | enolase-phosphatase E-1 | 1835045 | G | GC | 76 | 76 | 1 | 653 | PA1685 |  |
| 2169348 | A | AG | 154 | 154 | 1 |  |  |  | 2169348 | A | AG | 72 | 72 | 1 |  |  |  |
| 2186927 | G | GC | 65 | 65 | 1 |  |  |  | 2186927 | G | GC | 62 | 62 | 1 |  |  |  |
| 2195457 | G | GC | 177 | 177 | 1 |  |  |  | 2195457 | G | GC | 73 | 73 | 1 |  |  |  |
| 2239555 | A | AG | 157 | 157 | 1 |  |  |  | 2239555 | A | AG | 52 | 52 | 1 |  |  |  |
| 2342110 | C | CT | 223 | 223 | 1 |  |  |  | 2342110 | C | CT | 114 | 114 | 1 |  |  |  |
| 2355771 | A | AG | 199 | 200 | 0.995 | 88 | PA2139 | hypothetical protein | 2355771 | A | AG | 57 | 57 | 1 | 88 | PA2139 |  |
| 2356681 | GC | G | 163 | 163 | 1 | 514 | PA2141 | hypothetical protein | 2356681 | GC | G | 97 | 97 | 1 | 514 | PA2141 |  |
| 2532046 | G | GC | 174 | 174 | 1 |  |  |  | 2532046 | G | GC | 75 | 75 | 1 |  |  |  |
| 2753522 | G | GC | 169 | 169 | 1 | 924 | PA2452 | hypothetical protein | 2753522 | G | GC | 72 | 72 | 1 | 924 | PA2452 |  |
| 2807693 | TCGGCCAGC | T | 188 | 188 | 1 | 225 | PA2492 | transcriptional regulator MexT | 2807693 | TCGGCCAGC | T | 80 | 80 | 1 | 225 | PA2492 |  |
| 3016844 | G | GC | 195 | 195 | 1 | 41 | PA2668 | hypothetical protein | 3016844 | G | GC | 88 | 88 | 1 | 41 | PA2668 |  |
| 3083196 | A | AG | 163 | 163 | 1 | 3347 | PA2727 | hypothetical protein | 3083196 | A | AG | 77 | 77 | 1 | 3347 | PA2727 |  |
| 3919508 | G | GC | 225 | 225 | 1 |  |  |  | 3919508 | G | GC | 76 | 76 | 1 |  |  |  |
| 4539468 | G | GC | 169 | 169 | 1 | 313 | PA4059 | hypothetical protein | 4539468 | G | GC | 82 | 82 | 1 | 313 | PA4059 |  |
| 4888194 | A | AG | 193 | 195 | 0.989744 | 2 | PA4360 | hypothetical protein | 4888194 | A | AG | 111 | 111 | 1 | 2 | PA4360 |  |
| 5033101 | G | GC | 134 | 134 | 1 | 1614 | PA4496 | probable binding protein component of ABC transporter | 5033101 | G | GC | 97 | 98 | 0.989796 | 1614 | PA4496 |  |
| 5071543 | AACTG | A | 264 | 267 | 0.988764 |  |  |  | 5071543 | AACTG | A | 118 | 118 | 1 |  |  |  |
| 5472415 | C | CG | 214 | 214 | 1 |  |  |  | 5472415 | C | CG | 102 | 102 | 1 |  |  |  |
| 5655220 | C | CCGG | 170 | 171 | 0.994152 | 410 | PA5024 | conserved hypothetical protein | 5655220 | C | CCGG | 81 | 83 | 0.975904 | 410 | PA5024 |  |
|  |  |  |  |  |  |  |  |  |  |  |  |  |  |  |  |  |  |
| **sup3** | | | | | | | | | ***Δpa0947*(pTS-*pa0947*)** | | | | | | | | |
| **POS** | **REF** | **ALT** | **alt-coverage** | **all-coverage** | **alt-precent** | **Gene_loci** | **Gene_ID** | **function** | **POS** | **REF** | **ALT** | **alt-coverage** | **all-coverage** | **alt-precent** | **Gene_loci** | **Gene_ID** |  |
| 169283 | CG | C | 192 | 192 | 1 |  |  |  | 169283 | CG | C | 98 | 98 | 1 |  |  |  |
| 411125 | AC | A | 142 | 142 | 1 |  |  |  | 411125 | AC | A | 104 | 104 | 1 |  |  |  |
| 667028 | G | GC | 121 | 121 | 1 |  |  |  | 667028 | G | GC | 104 | 104 | 1 |  |  |  |
| 740419 | G | GC | 163 | 163 | 1 | 217 | PA0683 | probable type II secretion system protein | 740419 | G | GC | 90 | 90 | 1 | 217 | PA0683 |  |
| 816529 | G | GC | 155 | 155 | 1 |  |  |  | 816529 | G | GC | 102 | 102 | 1 |  |  |  |
| 891099 | A | AC | 119 | 119 | 1 |  |  |  | 891099 | A | AC | 91 | 91 | 1 |  |  |  |
| 1116213 | G | GC | 188 | 188 | 1 | 1 | PA1029 | hypothetical protein | 1116213 | G | GC | 104 | 104 | 1 | 1 | PA1029 |  |
| 1215657 | A | AG | 169 | 169 | 1 | 374 | PA1122 | probable peptide deformylase | 1215657 | A | AG | 97 | 97 | 1 | 374 | PA1122 |  |
| 1275766 | GA | G | 126 | 127 | 0.992126 | 33 | PA1174 | periplasmic nitrate reductase protein NapA | 1275766 | GA | G | 105 | 105 | 1 | 33 | PA1174 |  |
| 1440622 | CA | C | 115 | 115 | 1 | 1917 | PA1327 | probable protease | 1440622 | CA | C | 85 | 85 | 1 | 1917 | PA1327 |  |
| 1445357 | A | AG | 159 | 159 | 1 |  |  |  | 1445357 | A | AG | 65 | 65 | 1 |  |  |  |
| 1467482 | A | AG | 127 | 127 | 1 |  |  |  | 1467482 | A | AG | 96 | 96 | 1 |  |  |  |
| 1467483 | C | CG | 129 | 129 | 1 |  |  |  | 1467483 | C | CG | 98 | 98 | 1 |  |  |  |
| 1835045 | G | GC | 129 | 129 | 1 | 653 | PA1685 | enolase-phosphatase E-1 | 1835045 | G | GC | 76 | 76 | 1 | 653 | PA1685 |  |
| 2169348 | A | AG | 140 | 140 | 1 |  |  |  | 2169348 | A | AG | 72 | 72 | 1 |  |  |  |
| 2186927 | G | GC | 73 | 73 | 1 |  |  |  | 2186927 | G | GC | 62 | 62 | 1 |  |  |  |
| 2195457 | G | GC | 173 | 174 | 0.994253 |  |  |  | 2195457 | G | GC | 73 | 73 | 1 |  |  |  |
| 2239555 | A | AG | 164 | 164 | 1 |  |  |  | 2239555 | A | AG | 52 | 52 | 1 |  |  |  |
| 2342110 | C | CT | 212 | 212 | 1 |  |  |  | 2342110 | C | CT | 114 | 114 | 1 |  |  |  |
| 2355771 | A | AG | 172 | 172 | 1 | 88 | PA2139 | hypothetical protein | 2355771 | A | AG | 57 | 57 | 1 | 88 | PA2139 |  |
| 2356681 | GC | G | 140 | 140 | 1 | 514 | PA2141 | hypothetical protein | 2356681 | GC | G | 97 | 97 | 1 | 514 | PA2141 |  |
| 2532046 | G | GC | 153 | 153 | 1 |  |  |  | 2532046 | G | GC | 75 | 75 | 1 |  |  |  |
| 2753522 | G | GC | 162 | 162 | 1 | 924 | PA2452 | hypothetical protein | 2753522 | G | GC | 72 | 72 | 1 | 924 | PA2452 |  |
| 2807693 | TCGGCCAGC | T | 168 | 168 | 1 | 225 | PA2492 | transcriptional regulator MexT | 2807693 | TCGGCCAGC | T | 80 | 80 | 1 | 225 | PA2492 |  |
| 3016844 | G | GC | 168 | 168 | 1 | 41 | PA2668 | hypothetical protein | 3016844 | G | GC | 88 | 88 | 1 | 41 | PA2668 |  |
| 3083196 | A | AG | 139 | 139 | 1 | 3347 | PA2727 | hypothetical protein | 3083196 | A | AG | 77 | 77 | 1 | 3347 | PA2727 |  |
| 3919508 | G | GC | 189 | 189 | 1 |  |  |  | 3919508 | G | GC | 76 | 76 | 1 |  |  |  |
| 4539468 | G | GC | 160 | 160 | 1 | 313 | PA4059 | hypothetical protein | 4539468 | G | GC | 82 | 82 | 1 | 313 | PA4059 |  |
| 4888194 | A | AG | 150 | 150 | 1 | 2 | PA4360 | hypothetical protein | 4888194 | A | AG | 111 | 111 | 1 | 2 | PA4360 |  |
| 5033101 | G | GC | 123 | 123 | 1 | 1614 | PA4496 | probable binding protein component of ABC transporter | 5033101 | G | GC | 97 | 98 | 0.989796 | 1614 | PA4496 |  |
| 5071543 | AACTG | A | 254 | 254 | 1 |  |  |  | 5071543 | AACTG | A | 118 | 118 | 1 |  |  |  |
| 5472415 | C | CG | 222 | 223 | 0.995516 |  |  |  | 5472415 | C | CG | 102 | 102 | 1 |  |  |  |
| 5655220 | C | CCGG | 159 | 159 | 1 | 410 | PA5024 | conserved hypothetical protein | 5655220 | C | CCGG | 81 | 83 | 0.975904 | 410 | PA5024 |  |
|  |  |  |  |  |  |  |  |  |  |  |  |  |  |  |  |  |  |
| **sup4** | | | | | | | | | ***Δpa0947*(pTS-*pa0947*)** | | | | | | | |  |
| **POS** | **REF** | **ALT** | **alt-coverage** | **all-coverage** | **alt-precent** | **Gene_loci** | **Gene_ID** | **function** | **POS** | **REF** | **ALT** | **alt-coverage** | **all-coverage** | **alt-precent** | **Gene_loci** | **Gene_ID** |  |
| 169283 | CG | C | 97 | 97 | 1 |  |  |  | 169283 | CG | C | 98 | 98 | 1 |  |  |  |
| 411125 | AC | A | 95 | 95 | 1 |  |  |  | 411125 | AC | A | 104 | 104 | 1 |  |  |  |
| 667028 | G | GC | 101 | 101 | 1 |  |  |  | 667028 | G | GC | 104 | 104 | 1 |  |  |  |
| 740419 | G | GC | 133 | 133 | 1 | 217 | PA0683 | probable type II secretion system protein | 740419 | G | GC | 90 | 90 | 1 | 217 | PA0683 |  |
| 816529 | G | GC | 138 | 138 | 1 |  |  |  | 816529 | G | GC | 102 | 102 | 1 |  |  |  |
| 891099 | A | AC | 100 | 100 | 1 |  |  |  | 891099 | A | AC | 91 | 91 | 1 |  |  |  |
| 1116213 | G | GC | 176 | 176 | 1 | 1 | PA1029 | hypothetical protein | 1116213 | G | GC | 104 | 104 | 1 | 1 | PA1029 |  |
| 1215657 | A | AG | 144 | 144 | 1 | 374 | PA1122 | probable peptide deformylase | 1215657 | A | AG | 97 | 97 | 1 | 374 | PA1122 |  |
| 1275766 | GA | G | 101 | 101 | 1 | 33 | PA1174 | periplasmic nitrate reductase protein NapA | 1275766 | GA | G | 105 | 105 | 1 | 33 | PA1174 |  |
| 1440622 | CA | C | 105 | 108 | 0.972222 | 1917 | PA1327 | probable protease | 1440622 | CA | C | 85 | 85 | 1 | 1917 | PA1327 |  |
| 1445357 | A | AG | 150 | 150 | 1 |  |  |  | 1445357 | A | AG | 65 | 65 | 1 |  |  |  |
| 1467482 | A | AG | 111 | 111 | 1 |  |  |  | 1467482 | A | AG | 96 | 96 | 1 |  |  |  |
| 1467483 | C | CG | 111 | 111 | 1 |  |  |  | 1467483 | C | CG | 98 | 98 | 1 |  |  |  |
| 1835045 | G | GC | 150 | 150 | 1 | 653 | PA1685 | enolase-phosphatase E-1 | 1835045 | G | GC | 76 | 76 | 1 | 653 | PA1685 |  |
| 2169348 | A | AG | 138 | 138 | 1 |  |  |  | 2169348 | A | AG | 72 | 72 | 1 |  |  |  |
| 2186927 | G | GC | 62 | 62 | 1 |  |  |  | 2186927 | G | GC | 62 | 62 | 1 |  |  |  |
| 2195457 | G | GC | 168 | 168 | 1 |  |  |  | 2195457 | G | GC | 73 | 73 | 1 |  |  |  |
| 2239555 | A | AG | 120 | 120 | 1 |  |  |  | 2239555 | A | AG | 52 | 52 | 1 |  |  |  |
| 2342110 | C | CT | 170 | 170 | 1 |  |  |  | 2342110 | C | CT | 114 | 114 | 1 |  |  |  |
| 2355771 | A | AG | 188 | 188 | 1 | 88 | PA2139 | hypothetical protein | 2355771 | A | AG | 57 | 57 | 1 | 88 | PA2139 |  |
| 2356681 | GC | G | 137 | 137 | 1 | 514 | PA2141 | hypothetical protein | 2356681 | GC | G | 97 | 97 | 1 | 514 | PA2141 |  |
| 2532046 | G | GC | 125 | 125 | 1 |  |  |  | 2532046 | G | GC | 75 | 75 | 1 |  |  |  |
| 2753522 | G | GC | 135 | 135 | 1 | 924 | PA2452 | hypothetical protein | 2753522 | G | GC | 72 | 72 | 1 | 924 | PA2452 |  |
| 2807693 | TCGGCCAGC | T | 123 | 123 | 1 | 225 | PA2492 | transcriptional regulator MexT | 2807693 | TCGGCCAGC | T | 80 | 80 | 1 | 225 | PA2492 |  |
| 3016844 | G | GC | 172 | 172 | 1 | 41 | PA2668 | hypothetical protein | 3016844 | G | GC | 88 | 88 | 1 | 41 | PA2668 |  |
| 3083196 | A | AG | 122 | 122 | 1 | 3347 | PA2727 | hypothetical protein | 3083196 | A | AG | 77 | 77 | 1 | 3347 | PA2727 |  |
| 3919508 | G | GC | 181 | 181 | 1 |  |  |  | 3919508 | G | GC | 76 | 76 | 1 |  |  |  |
| 4539468 | G | GC | 100 | 100 | 1 | 313 | PA4059 | hypothetical protein | 4539468 | G | GC | 82 | 82 | 1 | 313 | PA4059 |  |
| 4888194 | A | AG | 136 | 136 | 1 | 2 | PA4360 | hypothetical protein | 4888194 | A | AG | 111 | 111 | 1 | 2 | PA4360 |  |
| 5033101 | G | GC | 107 | 107 | 1 | 1614 | PA4496 | probable binding protein component of ABC transporter | 5033101 | G | GC | 97 | 98 | 0.989796 | 1614 | PA4496 |  |
| 5071543 | AACTG | A | 212 | 212 | 1 |  |  |  | 5071543 | AACTG | A | 118 | 118 | 1 |  |  |  |
| 5472415 | C | CG | 149 | 149 | 1 |  |  |  | 5472415 | C | CG | 102 | 102 | 1 |  |  |  |
| 5655220 | C | CCGG | 129 | 129 | 1 | 410 | PA5024 | conserved hypothetical protein | 5655220 | C | CCGG | 81 | 83 | 0.975904 | 410 | PA5024 |  |
|  |  |  |  |  |  |  |  |  |  |  |  |  |  |  |  |  |  |
| **(D) SNP loci in both sup1-4 and *Δpa0947*(pTS-*pa0947*)** | | | | |  |  |  |  |  |  |  |  |  |  |  |  |  |
| **sup1** | | | | | | | | | ***Δpa0947*(pTS-*pa0947*)** | | | | | | | |  |
| **POS** | **REF** | **ALT** | **alt-coverage** | **all-coverage** | **alt-precent** | **Gene_loci** | **Gene_ID** | **function** | **POS** | **REF** | **ALT** | **alt-coverage** | **all-coverage** | **alt-precent** | **Gene_loci** | **Gene_ID** | **function** |
| 183697 | T | G | 170 | 170 | 1 | 930 | PA0159 | probable transcriptional regulator | 183697 | T | G | 82 | 82 | 1 | 930 | PA0159 | probable transcriptional regulator |
| 413850 | T | C | 138 | 138 | 1 |  |  |  | 413850 | T | C | 89 | 89 | 1 |  |  |  |
| 721611 | C | T | 65 | 65 | 1 |  |  |  | 721611 | C | T | 68 | 68 | 1 |  |  |  |
| 721622 | C | T | 63 | 63 | 1 |  |  |  | 721622 | C | T | 67 | 67 | 1 |  |  |  |
| 721663 | T | C | 47 | 47 | 1 |  |  |  | 721663 | T | C | 47 | 47 | 1 |  |  |  |
| 721664 | C | T | 46 | 46 | 1 |  |  |  | 721664 | C | T | 46 | 46 | 1 |  |  |  |
| 721667 | A | G | 43 | 43 | 1 |  |  |  | 721667 | A | G | 44 | 44 | 1 |  |  |  |
| 721670 | A | G | 44 | 44 | 1 |  |  |  | 721670 | A | G | 43 | 43 | 1 |  |  |  |
| 721718 | A | G | 39 | 39 | 1 |  |  |  | 721718 | A | G | 26 | 26 | 1 |  |  |  |
| 721725 | C | T | 37 | 37 | 1 |  |  |  | 721725 | C | T | 25 | 25 | 1 |  |  |  |
| 721740 | C | T | 37 | 37 | 1 |  |  |  | 721740 | C | T | 22 | 22 | 1 |  |  |  |
| 1589438 | G | C | 77 | 77 | 1 | 101 | PA1459 | probable methyltransferase | 1589438 | G | C | 101 | 101 | 1 | 101 | PA1459 | probable methyltransferase |
| 2239547 | T | G | 63 | 63 | 1 |  |  |  | 2239547 | T | G | 55 | 55 | 1 |  |  |  |
| 2669175 | G | C | 67 | 67 | 1 | 2455 | PA2400 | PvdJ | 2669175 | G | C | 66 | 66 | 1 | 2455 | PA2400 | PvdJ |
| 3133496 | G | T | 76 | 76 | 1 |  |  |  | 2807982 | T | A | 67 | 67 | 1 | 514 | PA2492 | transcriptional regulator MexT |
| 4212201 | A | G | 89 | 89 | 1 | 1907 | PA3760 | N-Acetyl-D-Glucosamine phosphotransferase system transporter | 4212201 | A | G | 85 | 85 | 1 | 1907 | PA3760 | N-Acetyl-D-Glucosamine phosphotransferase system transporter |
| 4344266 | A | G | 97 | 97 | 1 | 570 | PA3877 | nitrite extrusion protein 1 | 4344266 | A | G | 90 | 90 | 1 | 570 | PA3877 | nitrite extrusion protein 1 |
| 4448855 | C | G | 109 | 109 | 1 |  |  |  | 4448855 | C | G | 112 | 112 | 1 |  |  |  |
| 4448856 | G | C | 108 | 108 | 1 |  |  |  | 4448856 | G | C | 113 | 113 | 1 |  |  |  |
| 4869855 | T | G | 72 | 72 | 1 | 474 | PA4341 | probable transcriptional regulator | 4869855 | T | G | 96 | 96 | 1 | 474 | PA4341 | probable transcriptional regulator |
| 4924552 | C | G | 111 | 111 | 1 | 532 | PA4394 | conserved hypothetical protein | 4924552 | C | G | 111 | 111 | 1 | 532 | PA4394 | conserved hypothetical protein |
| 4924553 | G | C | 112 | 112 | 1 | 531 | PA4394 | conserved hypothetical protein | 4924553 | G | C | 109 | 109 | 1 | 531 | PA4394 | conserved hypothetical protein |
| 5036891 | A | C | 129 | 129 | 1 |  |  |  | 5036891 | A | C | 110 | 110 | 1 |  |  |  |
| 5743461 | C | G | 129 | 129 | 1 | 1293 | PA5100 | urocanase | 5743461 | C | G | 74 | 74 | 1 | 1293 | PA5100 | urocanase |
| 5743462 | G | C | 129 | 129 | 1 | 1292 | PA5100 | urocanase | 5743462 | G | C | 74 | 74 | 1 | 1292 | PA5100 | urocanase |
| 6079222 | A | G | 154 | 154 | 1 | 1179 | PA5399 | DgcB, Dimethylglycine catabolism | 6079222 | A | G | 81 | 81 | 1 | 1179 | PA5399 | DgcB, Dimethylglycine catabolism |
| 6098781 | G | C | 146 | 146 | 1 | 1758 | PA5418 | sarcosine oxidase alpha subunit | 6098781 | G | C | 124 | 124 | 1 | 1758 | PA5418 | sarcosine oxidase alpha subunit |
| 6115455 | T | G | 137 | 137 | 1 | 858 | PA5434 | tryptophan permease | 6115455 | T | G | 121 | 121 | 1 | 858 | PA5434 | tryptophan permease |
|  |  |  |  |  |  |  |  |  |  |  |  |  |  |  |  |  |  |
| **sup2** | | | | | | | | | ***Δpa0947*(pTS-*pa0947*)** | | | | | | | |  |
| **POS** | **REF** | **ALT** | **alt-coverage** | **all-coverage** | **alt-precent** | **Gene_loci** | **Gene_ID** | **function** | **POS** | **REF** | **ALT** | **alt-coverage** | **all-coverage** | **alt-precent** | **Gene_loci** | **Gene_ID** |  |
| 183697 | T | G | 322 | 322 | 1 | 930 | PA0159 | probable transcriptional regulator | 183697 | T | G | 82 | 82 | 1 | 930 | PA0159 |  |
| 413850 | T | C | 191 | 191 | 1 |  |  |  | 413850 | T | C | 89 | 89 | 1 |  |  |  |
| 721611 | C | T | 168 | 169 | 0.994083 |  |  |  | 721611 | C | T | 68 | 68 | 1 |  |  |  |
| 721622 | C | T | 161 | 161 | 1 |  |  |  | 721622 | C | T | 67 | 67 | 1 |  |  |  |
| 721663 | T | C | 90 | 90 | 1 |  |  |  | 721663 | T | C | 47 | 47 | 1 |  |  |  |
| 721664 | C | T | 88 | 88 | 1 |  |  |  | 721664 | C | T | 46 | 46 | 1 |  |  |  |
| 721667 | A | G | 83 | 83 | 1 |  |  |  | 721667 | A | G | 44 | 44 | 1 |  |  |  |
| 721670 | A | G | 78 | 78 | 1 |  |  |  | 721670 | A | G | 43 | 43 | 1 |  |  |  |
| 721718 | A | G | 50 | 50 | 1 |  |  |  | 721718 | A | G | 26 | 26 | 1 |  |  |  |
| 721725 | C | T | 46 | 46 | 1 |  |  |  | 721725 | C | T | 25 | 25 | 1 |  |  |  |
| 721740 | C | T | 38 | 38 | 1 |  |  |  | 721740 | C | T | 22 | 22 | 1 |  |  |  |
| 1589438 | G | C | 227 | 227 | 1 | 101 | PA1459 | probable methyltransferase | 1589438 | G | C | 101 | 101 | 1 | 101 | PA1459 |  |
| 2239547 | T | G | 152 | 152 | 1 |  |  |  | 2239547 | T | G | 55 | 55 | 1 |  |  |  |
| 2669175 | G | C | 182 | 182 | 1 | 2455 | PA2400 | PvdJ | 2669175 | G | C | 66 | 66 | 1 | 2455 | PA2400 |  |
| 2807982 | T | A | 166 | 166 | 1 | 514 | PA2492 | transcriptional regulator MexT | 2807982 | T | A | 67 | 67 | 1 | 514 | PA2492 |  |
| 4212201 | A | G | 208 | 209 | 0.995215 | 1907 | PA3760 | N-Acetyl-D-Glucosamine phosphotransferase system transporter | 4212201 | A | G | 85 | 85 | 1 | 1907 | PA3760 |  |
| 4344266 | A | G | 239 | 239 | 1 | 570 | PA3877 | nitrite extrusion protein 1 | 4344266 | A | G | 90 | 90 | 1 | 570 | PA3877 |  |
| 4448855 | C | G | 135 | 138 | 0.978261 |  |  |  | 4448855 | C | G | 112 | 112 | 1 |  |  |  |
| 4448856 | G | C | 138 | 141 | 0.978723 |  |  |  | 4448856 | G | C | 113 | 113 | 1 |  |  |  |
| 4869855 | T | G | 180 | 180 | 1 | 474 | PA4341 | probable transcriptional regulator | 4869855 | T | G | 96 | 96 | 1 | 474 | PA4341 |  |
| 4924552 | C | G | 217 | 217 | 1 | 532 | PA4394 | conserved hypothetical protein | 4924552 | C | G | 111 | 111 | 1 | 532 | PA4394 |  |
| 4924553 | G | C | 217 | 217 | 1 | 531 | PA4394 | conserved hypothetical protein | 4924553 | G | C | 109 | 109 | 1 | 531 | PA4394 |  |
| 5036891 | A | C | 286 | 286 | 1 |  |  |  | 5036891 | A | C | 110 | 110 | 1 |  |  |  |
| 5743461 | C | G | 207 | 207 | 1 | 1293 | PA5100 | urocanase | 5743461 | C | G | 74 | 74 | 1 | 1293 | PA5100 |  |
| 5743462 | G | C | 207 | 207 | 1 | 1292 | PA5100 | urocanase | 5743462 | G | C | 74 | 74 | 1 | 1292 | PA5100 |  |
| 6079222 | A | G | 236 | 236 | 1 | 1179 | PA5399 | DgcB, Dimethylglycine catabolism | 6079222 | A | G | 81 | 81 | 1 | 1179 | PA5399 |  |
| 6098781 | G | C | 254 | 255 | 0.996078 | 1758 | PA5418 | sarcosine oxidase alpha subunit | 6098781 | G | C | 124 | 124 | 1 | 1758 | PA5418 |  |
| 6115455 | T | G | 300 | 300 | 1 | 858 | PA5434 | tryptophan permease | 6115455 | T | G | 121 | 121 | 1 | 858 | PA5434 |  |
|  |  |  |  |  |  |  |  |  |  |  |  |  |  |  |  |  |  |
| **sup3** | | | | | | | | | ***Δpa0947*(pTS-*pa0947*)** | | | | | | | |  |
| **POS** | **REF** | **ALT** | **alt-coverage** | **all-coverage** | **alt-precent** | **Gene_loci** | **Gene_ID** | **function** | **POS** | **REF** | **ALT** | **alt-coverage** | **all-coverage** | **alt-precent** | **Gene_loci** | **Gene_ID** |  |
| 183697 | T | G | 364 | 364 | 1 | 930 | PA0159 | probable transcriptional regulator | 183697 | T | G | 82 | 82 | 1 | 930 | PA0159 |  |
| 413850 | T | C | 153 | 153 | 1 |  |  |  | 413850 | T | C | 89 | 89 | 1 |  |  |  |
| 721611 | C | T | 146 | 147 | 0.993197 |  |  |  | 721611 | C | T | 68 | 68 | 1 |  |  |  |
| 721622 | C | T | 135 | 136 | 0.992647 |  |  |  | 721622 | C | T | 67 | 67 | 1 |  |  |  |
| 721663 | T | C | 88 | 89 | 0.988764 |  |  |  | 721663 | T | C | 47 | 47 | 1 |  |  |  |
| 721664 | C | T | 86 | 87 | 0.988506 |  |  |  | 721664 | C | T | 46 | 46 | 1 |  |  |  |
| 721667 | A | G | 82 | 83 | 0.987952 |  |  |  | 721667 | A | G | 44 | 44 | 1 |  |  |  |
| 721670 | A | G | 78 | 79 | 0.987342 |  |  |  | 721670 | A | G | 43 | 43 | 1 |  |  |  |
| 721718 | A | G | 51 | 51 | 1 |  |  |  | 721718 | A | G | 26 | 26 | 1 |  |  |  |
| 721725 | C | T | 51 | 51 | 1 |  |  |  | 721725 | C | T | 25 | 25 | 1 |  |  |  |
| 721740 | C | T | 48 | 48 | 1 |  |  |  | 721740 | C | T | 22 | 22 | 1 |  |  |  |
| 1589438 | G | C | 168 | 168 | 1 | 101 | PA1459 | probable methyltransferase | 1589438 | G | C | 101 | 101 | 1 | 101 | PA1459 |  |
| 2239547 | T | G | 159 | 159 | 1 |  |  |  | 2239547 | T | G | 55 | 55 | 1 |  |  |  |
| 2669175 | G | C | 158 | 158 | 1 | 2455 | PA2400 | PvdJ | 2669175 | G | C | 66 | 66 | 1 | 2455 | PA2400 |  |
| 2807982 | T | A | 127 | 127 | 1 | 514 | PA2492 | transcriptional regulator MexT | 2807982 | T | A | 67 | 67 | 1 | 514 | PA2492 |  |
| 4212201 | A | G | 148 | 148 | 1 | 1907 | PA3760 | N-Acetyl-D-Glucosamine phosphotransferase system transporter | 4212201 | A | G | 85 | 85 | 1 | 1907 | PA3760 |  |
| 4344266 | A | G | 173 | 173 | 1 | 570 | PA3877 | nitrite extrusion protein 1 | 4344266 | A | G | 90 | 90 | 1 | 570 | PA3877 |  |
| 4448855 | C | G | 150 | 150 | 1 |  |  |  | 4448855 | C | G | 112 | 112 | 1 |  |  |  |
| 4448856 | G | C | 148 | 148 | 1 |  |  |  | 4448856 | G | C | 113 | 113 | 1 |  |  |  |
| 4869855 | T | G | 134 | 134 | 1 | 474 | PA4341 | probable transcriptional regulator | 4869855 | T | G | 96 | 96 | 1 | 474 | PA4341 |  |
| 4924552 | C | G | 183 | 183 | 1 | 532 | PA4394 | conserved hypothetical protein | 4924552 | C | G | 111 | 111 | 1 | 532 | PA4394 |  |
| 4924553 | G | C | 185 | 185 | 1 | 531 | PA4394 | conserved hypothetical protein | 4924553 | G | C | 109 | 109 | 1 | 531 | PA4394 |  |
| 5036891 | A | C | 225 | 225 | 1 |  |  |  | 5036891 | A | C | 110 | 110 | 1 |  |  |  |
| 5743461 | C | G | 186 | 186 | 1 | 1293 | PA5100 | urocanase | 5743461 | C | G | 74 | 74 | 1 | 1293 | PA5100 |  |
| 5743462 | G | C | 185 | 185 | 1 | 1292 | PA5100 | urocanase | 5743462 | G | C | 74 | 74 | 1 | 1292 | PA5100 |  |
| 6079222 | A | G | 196 | 196 | 1 | 1179 | PA5399 | DgcB, Dimethylglycine catabolism | 6079222 | A | G | 81 | 81 | 1 | 1179 | PA5399 |  |
| 6098781 | G | C | 220 | 220 | 1 | 1758 | PA5418 | sarcosine oxidase alpha subunit | 6098781 | G | C | 124 | 124 | 1 | 1758 | PA5418 |  |
| 6115455 | T | G | 269 | 270 | 0.996296 | 858 | PA5434 | tryptophan permease | 6115455 | T | G | 121 | 121 | 1 | 858 | PA5434 |  |
|  |  |  |  |  |  |  |  |  |  |  |  |  |  |  |  |  |  |
| **sup4** | | | | | | | | | ***Δpa0947*(pTS-*pa0947*)** | | | | | | | |  |
| **POS** | **REF** | **ALT** | **alt-coverage** | **all-coverage** | **alt-precent** | **Gene_loci** | **Gene_ID** | **function** | **POS** | **REF** | **ALT** | **alt-coverage** | **all-coverage** | **alt-precent** | **Gene_loci** | **Gene_ID** |  |
| 183697 | T | G | 236 | 236 | 1 | 930 | PA0159 | probable transcriptional regulator | 183697 | T | G | 82 | 82 | 1 | 930 | PA0159 |  |
| 413850 | T | C | 127 | 127 | 1 |  |  |  | 413850 | T | C | 89 | 89 | 1 |  |  |  |
| 721611 | C | T | 126 | 126 | 1 |  |  |  | 721611 | C | T | 68 | 68 | 1 |  |  |  |
| 721622 | C | T | 120 | 120 | 1 |  |  |  | 721622 | C | T | 67 | 67 | 1 |  |  |  |
| 721663 | T | C | 72 | 72 | 1 |  |  |  | 721663 | T | C | 47 | 47 | 1 |  |  |  |
| 721664 | C | T | 70 | 70 | 1 |  |  |  | 721664 | C | T | 46 | 46 | 1 |  |  |  |
| 721667 | A | G | 70 | 70 | 1 |  |  |  | 721667 | A | G | 44 | 44 | 1 |  |  |  |
| 721670 | A | G | 63 | 63 | 1 |  |  |  | 721670 | A | G | 43 | 43 | 1 |  |  |  |
| 721718 | A | G | 49 | 49 | 1 |  |  |  | 721718 | A | G | 26 | 26 | 1 |  |  |  |
| 721725 | C | T | 48 | 48 | 1 |  |  |  | 721725 | C | T | 25 | 25 | 1 |  |  |  |
| 721740 | C | T | 43 | 43 | 1 |  |  |  | 721740 | C | T | 22 | 22 | 1 |  |  |  |
| 1589438 | G | C | 190 | 190 | 1 | 101 | PA1459 | probable methyltransferase | 1589438 | G | C | 101 | 101 | 1 | 101 | PA1459 |  |
| 2239547 | T | G | 129 | 129 | 1 |  |  |  | 2239547 | T | G | 55 | 55 | 1 |  |  |  |
| 2669175 | G | C | 173 | 173 | 1 | 2455 | PA2400 | PvdJ | 2669175 | G | C | 66 | 66 | 1 | 2455 | PA2400 |  |
| 2807982 | T | A | 184 | 184 | 1 | 514 | PA2492 | transcriptional regulator MexT | 2807982 | T | A | 67 | 67 | 1 | 514 | PA2492 |  |
| 4212201 | A | G | 177 | 177 | 1 | 1907 | PA3760 | N-Acetyl-D-Glucosamine phosphotransferase system transporter | 4212201 | A | G | 85 | 85 | 1 | 1907 | PA3760 |  |
| 4344266 | A | G | 166 | 166 | 1 | 570 | PA3877 | nitrite extrusion protein 1 | 4344266 | A | G | 90 | 90 | 1 | 570 | PA3877 |  |
| 4448855 | C | G | 107 | 107 | 1 |  |  |  | 4448855 | C | G | 112 | 112 | 1 |  |  |  |
| 4448856 | G | C | 106 | 106 | 1 |  |  |  | 4448856 | G | C | 113 | 113 | 1 |  |  |  |
| 4869855 | T | G | 132 | 132 | 1 | 474 | PA4341 | probable transcriptional regulator | 4869855 | T | G | 96 | 96 | 1 | 474 | PA4341 |  |
| 4924552 | C | G | 156 | 156 | 1 | 532 | PA4394 | conserved hypothetical protein | 4924552 | C | G | 111 | 111 | 1 | 532 | PA4394 |  |
| 4924553 | G | C | 157 | 157 | 1 | 531 | PA4394 | conserved hypothetical protein | 4924553 | G | C | 109 | 109 | 1 | 531 | PA4394 |  |
| 5036891 | A | C | 196 | 196 | 1 |  |  |  | 5036891 | A | C | 110 | 110 | 1 |  |  |  |
| 5743461 | C | G | 165 | 165 | 1 | 1293 | PA5100 | urocanase | 5743461 | C | G | 74 | 74 | 1 | 1293 | PA5100 |  |
| 5743462 | G | C | 166 | 166 | 1 | 1292 | PA5100 | urocanase | 5743462 | G | C | 74 | 74 | 1 | 1292 | PA5100 |  |
| 6079222 | A | G | 191 | 191 | 1 | 1179 | PA5399 | DgcB, Dimethylglycine catabolism | 6079222 | A | G | 81 | 81 | 1 | 1179 | PA5399 |  |
| 6098781 | G | C | 194 | 194 | 1 | 1758 | PA5418 | sarcosine oxidase alpha subunit | 6098781 | G | C | 124 | 124 | 1 | 1758 | PA5418 |  |
| 6115455 | T | G | 193 | 193 | 1 | 858 | PA5434 | tryptophan permease | 6115455 | T | G | 121 | 121 | 1 | 858 | PA5434 |  |
|  |  |  |  |  |  |  |  |  |  |  |  |  |  |  |  |  |  |
